# Epigenetic modifications are associated with inter-species gene expression variation in primates

**DOI:** 10.1186/s13059-014-0547-3

**Published:** 2014-12-03

**Authors:** Xiang Zhou, Carolyn E Cain, Marsha Myrthil, Noah Lewellen, Katelyn Michelini, Emily R Davenport, Matthew Stephens, Jonathan K Pritchard, Yoav Gilad

**Affiliations:** Department of Human Genetics, University of Chicago, Chicago, IL 60637 USA; Department of Statistics, University of Chicago, Chicago, IL 60637 USA; Howard Hughes Medical Institute, Chevy Chase, MD 20815 USA; Present address: Department of Biostatistics, University of Michigan, Ann Arbor, MI 48109 USA; Present address: Departments of Genetics and Biology, Stanford University, Stanford, CA 94305 USA

## Abstract

**Background:**

Changes in gene regulation have long been thought to play an important role in evolution and speciation, especially in primates. Over the past decade, comparative genomic studies have revealed extensive inter-species differences in gene expression levels, yet we know much less about the extent to which regulatory mechanisms differ between species.

**Results:**

To begin addressing this gap, we perform a comparative epigenetic study in primate lymphoblastoid cell lines, to query the contribution of RNA polymerase II and four histone modifications, H3K4me1, H3K4me3, H3K27ac, and H3K27me3, to inter-species variation in gene expression levels. We find that inter-species differences in mark enrichment near transcription start sites are significantly more often associated with inter-species differences in the corresponding gene expression level than expected by chance alone. Interestingly, we also find that first-order interactions among the five marks, as well as chromatin states, do not markedly contribute to the degree of association between the marks and inter-species variation in gene expression levels, suggesting that the marginal effects of the five marks dominate this contribution.

**Conclusions:**

Our observations suggest that epigenetic modifications are substantially associated with changes in gene expression levels among primates and may represent important molecular mechanisms in primate evolution.

**Electronic supplementary material:**

The online version of this article (doi:10.1186/s13059-014-0547-3) contains supplementary material, which is available to authorized users.

## Background

Differences in gene expression level have long been thought to underlie differences in phenotypes between species [1-4], and in particular, to contribute to adaptive evolution in primates [5,6]. Consistent with this, previous studies have identified a large number of genes differentially expressed among primates [7-16], and in a few cases, have also found that the inter-species changes in gene expression level might explain differences in complex phenotypes between primates [17-22]. However, we still know little about the underling regulatory mechanisms leading to the differences in gene expression levels across species. In particular, although a few studies have shown that the inter-species differences in certain epigenetic mechanisms can explain (in a statistical sense) a small proportion of variation in gene expression levels between species [23-25], the relative importance of evolutionary changes in different epigenetic regulatory mechanisms remains largely elusive.

The present study aims to take another step towards understanding gene regulatory evolution in primates, by focusing on inter-species differences in epigenetic regulatory mechanisms that are functionally associated with the regulation of transcription initiation. By studying a number of regulatory mechanisms in parallel in multiple primate species, we can assess the extent to which such differences are associated with inter-species variation in gene expression levels.

We focused on mechanisms associated with transcription initiation, a major determinant of overall steady-state gene expression levels [26-28]. Transcription of mRNA is preceded by the assembly of large protein complexes that coordinate the recruitment, initiation, and elongation of RNA polymerase II (Pol II) [29]. Assembly of these large protein complexes relies on epigenetic information, including various histone modifications [30], not only to provide an additional layer of targets for regulatory proteins, but also to directly affect chromatin accessibility of the promoter region to DNA-binding proteins [31]. As a result, Pol II occupancy and abundance of histone modifications are highly predictive of gene expression levels in multiple cell types [27,32-35].

A natural hypothesis is that inter-species variation in epigenetic modifications and Pol II abundance could in part contribute to gene expression differences between species. In support of this, a number of examples showed associations between the two. For instance, in *Arabidopsis* leaves, the enrichment of both H3K9ac and H3K4me3 in promoters is associated with transcript abundance between species [36]. During adipogenesis, orthologous genes with similar expression levels in mouse and human are often marked by similar histone modifications, and orthologous genes associated with inter-species differences in histone modifications are often differentially expressed between species [37]. In human, mouse, and pig pluripotent stem cells, the difference in the abundance of several histone modifications correlates with gene expression difference between species [38].

Recent comparative studies of certain epigenetic modifications in primates provide further support for the association between epigenetic modification variation and gene expression variation [23-25,39]. For example, Pai *et al.* showed that inter-species differences in DNA methylation pattern correlate with differences in gene expression level across species [24], and Cain *et al.* found that inter-species differences in the profile of the histone modification H3K4me3 are associated with changes in gene expression level between species [25]. However, the abundance difference in either of the two marks accounts for only a small proportion of gene expression difference between primates, and it remains unclear whether changes to epigenetic marks play a major role in regulatory evolution.

Here, we performed a comparative epigenetic study in primates to query the contribution of Pol II and four histone modifications (H3K4me1, H3K4me3, H3K27ac, and H3K27me3) to inter-species variation in gene expression levels. We choose these five marks not only because their molecular functions have been relatively well studied, but also because they represent a wide variety of transcription initiation regulators. In particular, the four histone modifications mark important regulatory regions: H3K4me1 is present at both active and poised enhancers [34,40-42], H3K4me3 marks active transcription start sites (TSSs) [34,43-45], H3K27ac marks active enhancers and promoters [32,46-48], and H3K27me3 marks repressed genomic regions [49,50]. In turn, Pol II directly interacts with chromatin remodeling factors [51] and catalyzes the transcription of mRNA [52].

In what follows, we evaluate the association of each of the five marks with gene expression level variation across species, and further, the joint contribution of all of them to the association with variation in gene expression, both within, but more importantly between, species.

## Results

### Genome-wide profiling of Pol II, four histone marks, and mRNA

We used chromatin immunoprecipitation (ChIP) followed by massively parallel sequencing (ChIPseq) to identify genomic regions associated with Pol II as well as with four histone modifications (H3K4me1, H3K4me3, H3K27ac, and H3K27me3) in lymphoblastoid cell lines (LCLs) from eight individuals from each of the three primate species, humans, chimpanzees, and rhesus macaques (a total of 24 samples for all marks except H3K27ac, for which a rhesus macaque sample is missing; Table S1 in Additional file 1; Additional file 2). We also extracted RNA from the same 24 LCLs and performed gene expression profiling in each sample by high-throughput sequencing (RNAseq; Table S1 in Additional file 1; Additional file 2).

As a first step of our analysis we used BWA [53] to align sequence reads to their respective reference genomes (human, hg19; chimpanzee, panTro3; rhesus macaque, rheMac2; Tables S2 to S4 in Additional file 1). Following convention, we then used RSEG [54] to identify enriched (broad) regions for H3K27me3 and used MACS [55] to identify (narrow) peaks for the other four marks (Tables S5 to S6 in Additional file 1). To minimize the number of falsely identified mark enrichment differences between species, we used two-step cutoffs to classify the enriched regions/peaks for each mark [25]. Our approach reflects the assumption that epigenetic profiles in orthologous regions will more often be shared than divergent. Briefly (see Materials and methods for more details), we first used a stringent cutoff to identify enriched regions with high confidence. Conditional on observing an enriched region in one individual using the stringent cutoff, we then classified the same or orthologous regions as enriched in other individuals with a more relaxed second cutoff (Additional file 3). Effectively, the more relaxed second threshold borrows information across species to increase power to detect enriched regions in any individual (regardless of species), and reduces the tendency to falsely detect differences in mark abundance between species. Once peak regions were identified, we obtained ‘normalized peak read’ counts for each individual by subtracting the number of mapped reads in the control sample from the number of mapped reads in the ChIPseq sample and further normalizing the resulting values to reads per kilobase per million mapped reads (RPKM) [56].

To facilitate comparisons between species that are focused on regions centered on expressed genes, we used liftOver [57] to identify orthologous TSSs and followed a previously described approach [16] to identify orthologous exons. We annotated orthologous TSSs and orthologous exons in a total of 26,115 genes. In order to analyze our data in a broader context, we considered 15 different chromatin state annotations previously identified in LCLs in the human genome [33,58]. We followed a previously published approach (of using liftOver [16]) to identify 308,514 orthologous regions with chromatin state annotations in all three genomes.

We confirmed that both the ChIPseq and RNAseq data are of high quality and that marks for individuals within each species are highly correlated (Additional file 4). Our chromatin marks data also show the expected enrichment pattern in the 15 chromatin states [33,58] across the genome. Specifically, H3K4me1 is enriched in strong and weak enhancers, H3K4me3 is enriched in promoters, H3K27ac is enriched in both promoters and enhancers, H3K27me3 is enriched in both poised promoters and repressed regions, while Pol II is enriched in strong promoters (Additional file 5).

### Pol II and four histone modifications are enriched near transcription start sites

**Figure 1:**
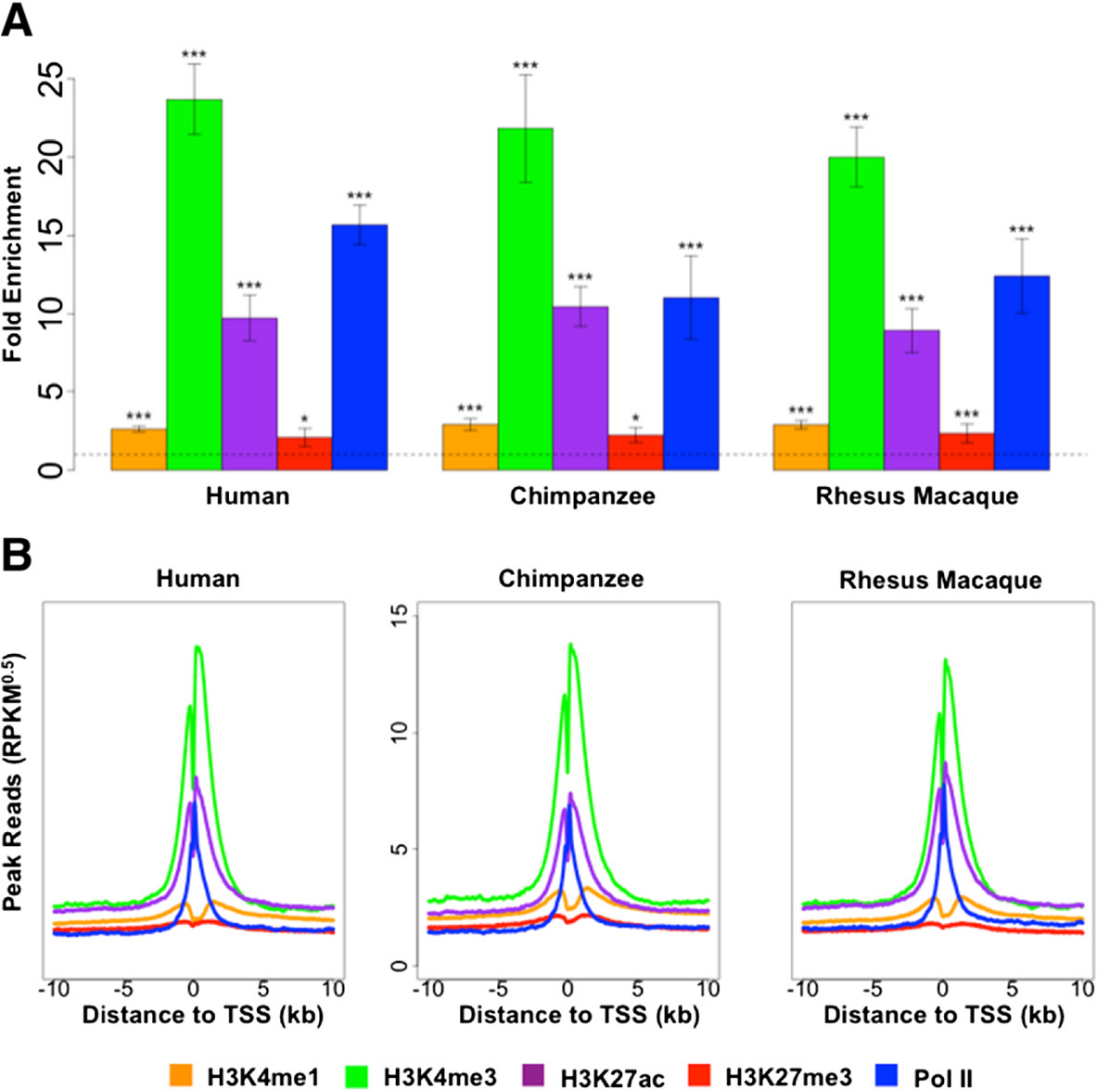
**Marks are enriched near transcription start sites. (A)** Fold enrichment of the five marks in ±2 kb regions near TSSs in the three primates. Error bars indicate standard deviation calculated across eight individuals in each species. Asterisks indicate significance levels based on Mann-Whitney one-sided tests (*P < 0.05, **P < 0.01, ***P < 0.001). **(B)** Distribution of normalized peak read counts for five marks around TSSs for each of the three primates. Units are in square root of RPKM (that is, RPKM^0.5^) and are averaged across individuals and across genes.

We expected the five marks (Pol II and four histone modifications) to be enriched near TSSs in all three primates, as has been shown previously in other contexts [25,27,35,38,50]. To examine this, we considered the average normalized peak read counts in ±2 kb regions near TSSs across all genes for each individual (more precisely, the regions begin at 2 kb upstream of the TSSs and end at the start of the second orthologous exon or 2 kb downstream of the TSSs, whichever is shorter). Similarly, for each individual, we obtained the normalized peak read counts over the entire genome. We then calculated fold enrichment in regions near TSSs for each mark by considering the ratio of these two values for each individual. We also performed non-parametric Mann-Whitney one-sided tests, based on data from all eight individuals in each species, to determine whether the normalized peak read counts in TSS regions are significantly higher than their genome-wide counterparts. The results of these analyses clearly indicate that all five marks are significantly enriched near TSSs, regardless of species (Figure 1A). The enrichment pattern is robust with respect to the choice of the size of the TSS region, but gradually decreases for increasingly larger regions around TSSs (Additional file 6).

To explore the localization pattern of the five marks near TSSs, we generated, for each species, the distributions of normalized peak read counts averaged across all genes and all individuals (Figure 1B). Consistent with previous studies [25,27,34,35,38,50,59], all five marks display bimodal distribution patterns near TSSs - albeit to a lesser extent for H3K27me3 - with two modes flanking the TSSs.

Levels of the five marks are also highly correlated with each other in regions near TSSs (Additional file 7). Specifically, H3K27me3 levels are negatively correlated with the other four marks, while H3K4me1, H3K4me3, H3K27ac and Pol II levels are positively correlated with each other.

### Mark abundance near transcription start sites correlates with gene expression levels within species

**Figure 2:**
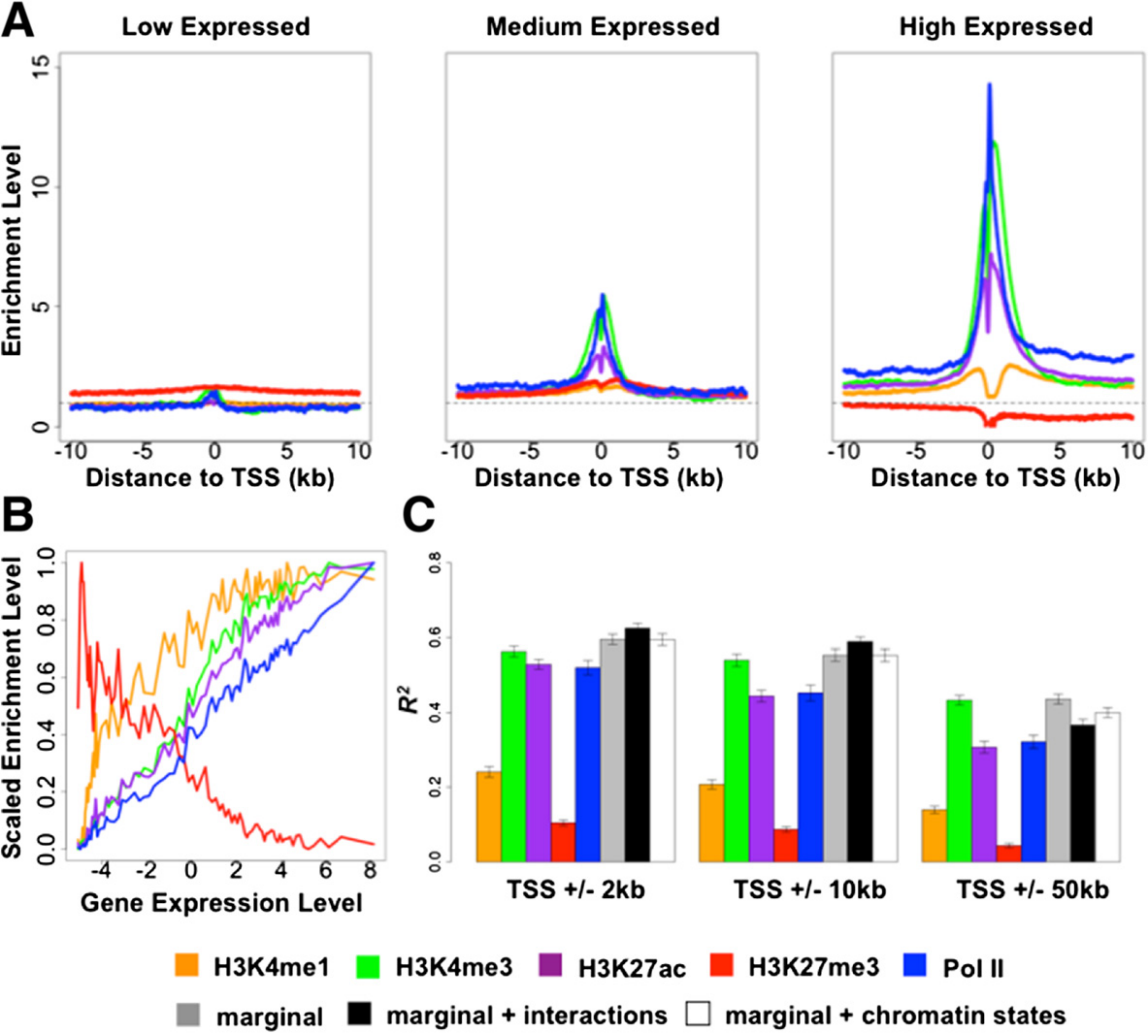
**Mark enrichment levels are correlated with gene expression levels in human. (A)** Density of enrichment level for five marks around TSSs for genes with low, medium, and high expression levels. Values are averaged across individuals and across genes in each category. **(B)** Mark enrichment levels plotted against gene expression levels for sliding windows of genes (n = 200) ordered from low to high expression levels. Enrichment levels are obtained in ±2 kb regions near TSSs and scaled to be between 0 and 1. All values are averaged across individuals and across genes in the window. **(C)** Proportion of variance in gene expression levels explained (R squared) by individual marginal effects (five colored bars), combined mark marginal effects (grey bars), all first-order interaction effects in addition to marginal effects (black bars), and all chromatin state-specific effects in addition to marginal effects (white bars) of the five marks. Results are shown for enrichment levels in TSS regions with increasing length. Error bars indicate standard deviation calculated based on 20 split replicates.

To explore the relationship between mark abundance and gene expression levels, we first obtained quantitative measurements and performed appropriate transformations for both mark enrichment level and RNA expression level (see Materials and methods for details). Next, we divided genes evenly (thus, arbitrarily) into the following three sets based on their expression levels: highly expressed, intermediately expressed and expressed at low levels. We obtained the distribution of the mark enrichment levels near TSSs, averaged across individuals within a species and across genes in each given set (Figure 2A; Figure S7A in Additional file 8; Figure S8A in Additional file 9). Regardless of species, we found that the repressive mark H3K27me3 [49,50] is enriched near TSSs of genes expressed at low levels, whereas Pol II and the other four active histone marks [32,34,40-48,52] are highly enriched near TSSs of highly expressed genes. To verify that these patterns are robust, we arbitrarily divided genes into a larger number of groups based on absolute gene expression levels, such that each group contains 200 genes (except the first group, which contains all non-expressed genes, and the last group, which contains fewer than 200 genes). We plotted the mean mark enrichment levels in the ±2 kb region near TSSs against the mean gene expression levels in each group, both averaged across individuals within a species and across genes in that group (Figure 2B; Figure S7B in Additional file 8; Figure S8B in Additional file 9; Additional file 10). We again observed a negative trend between the enrichment levels of H3K27me3 and gene expression levels, as well as positive trends for the correlations between the enrichment levels of the other four marks and gene expression levels. These trends were robust with respect to the choice of TSS region size (Additional file 10).

To quantitatively measure the relationship, namely the extent of association, between mark abundance and gene expression levels across genes within each species, we fitted a linear model for all genes, with gene expression level as response and mark enrichment level in regions near TSSs as covariates (averaged across individuals). In addition, to avoid model over-fitting, we used a 10-fold cross-validation (with 20 split replicates) and calculated R squared, in the test set (Figure 2C; Figure S7C in Additional file 8; Figure S8C in Additional file 9; Additional file 11). We found that the R squared by H3K4me3, H3K27ac, or Pol II is much higher than the R squared by the other two marks. Our observations with respect to individual marks are in close agreement with results from previous studies in other tissues [27,32,60]. In a statistical sense, levels of the five marks combined explain approximately 58% of the variance in gene expression levels within species (59% in human, 58% in chimpanzee, and 57% in rhesus macaque).

Because the marks show strong correlation patterns near TSSs (Additional file 7), and because previous studies have shown that combinatorial patterns of histone modifications and Pol II (that is, chromatin states) could be of biological importance [33,58], we asked if adding interaction effects increases the R squared. To do so, we considered all first-order interactions among marks - including all interactions between two marks, among three marks, and so on - in addition to their marginal effects. We used a Bayesian variable selection regression (BVSR) model [61-64] with gene expression level as response and all marginal and interaction terms as covariates. BVSR provides a 'posterior inclusion probability' (PIP) for each covariate, which indicates the confidence that the covariate contributes to prediction of phenotype. In addition, BVSR can produce reliable estimates of the proportion of variance explained by all covariates [61,64]. We used the posterior means as coefficient estimates and calculated R squared in the test set. Using this approach, we found that all marginal effects, except for H3K4me1, are important features that are consistently selected by the model (PIP >0.9; Additional file 12). Among the interaction features, interactions H3K4me1-H3K4me3 with or without Pol II, H3K4me1-H3K27ac with or without Pol II, H3K4me1-H3K27me3 with or without H3K4me3, H3K4me3-H3K27ac with or without Pol II, H3K27ac-Pol II are consistently selected as important features (PIP >0.9; Additional file 12). Somewhat surprisingly, however, considering all interaction features does not increase much the association of the marks with variation in gene expression levels across genes within species (black bars versus grey bars in Figure 2C; Figure S7C in Additional file 8; Figure S8C in Additional file 9).

To further explore the importance of mark combinatory patterns, we directly looked at state-specific mark effects with respect to the 15 different chromatin states near TSSs. Fitting a BVSR with both marginal effects and mark enrichment levels in the 15 chromatin states as covariates, we again found that all marginal effects, except for H3K27ac, are important features (Additional file 13). Among the mark enrichment levels in different chromatin states, H3K4me1 and H3K27ac in strong enhancers (state 4), as well as H3K4me1 and Pol II in repetitive regions (state 13 and state 14, respectively) are consistently selected as important features (Additional file 13), which is not unexpected given their importance in various interaction terms we identified when we considered our own data alone. Again, somewhat surprisingly, considering state-specific mark effects in all chromatin states does not explain much additional variance in gene expression levels within species (white bars versus grey bars in Figure 2C; Figure S7C in Additional file 8; Figure S8C in Additional file 9). In fact, considering chromatin states as far as 250 kb away from TSSs does not increase the explained variance (R squared are still 0.60 ± 0.01, 0.58 ± 0.01, 0.58 ± 0.01 in human, chimpanzee, and rhesus macaque, respectively).

### Differences in mark enrichment are associated with gene expression differences across species

Next, we considered differences between species. As a first step, we identified differentially expressed (DE) genes across species, as well as orthologous TSS regions that are associated with inter-species differences in enrichment of histone marks or Pol II. As expected, we found a smaller number of differences between humans and chimpanzees than between either humans or chimpanzees and rhesus macaques (Table 1; Tables S7 and S8 in Additional file 1; Additional file 14).

**Table 1:** **Number of transcription start site regions associated with interspecies differences in enrichment of marks and number of differentially expressed genes from pairwise comparisons among three primates at a false discovery rate cutoff of 5%**

	**H3K4me1**	**H3K4me3**	**H3K27ac**	**H3K27me3**	**Pol II**	**RNA**
H versus C	137	3,037	3,176	438	1,577	3,824
H versus R	3,298	5,257	5,549	1,487	3,708	6,567
C versus R	3,421	4,928	5,456	1,017	3,299	5,914

C, chimpanzee; H, human, R, rhesus macaque.

**Figure 3:**
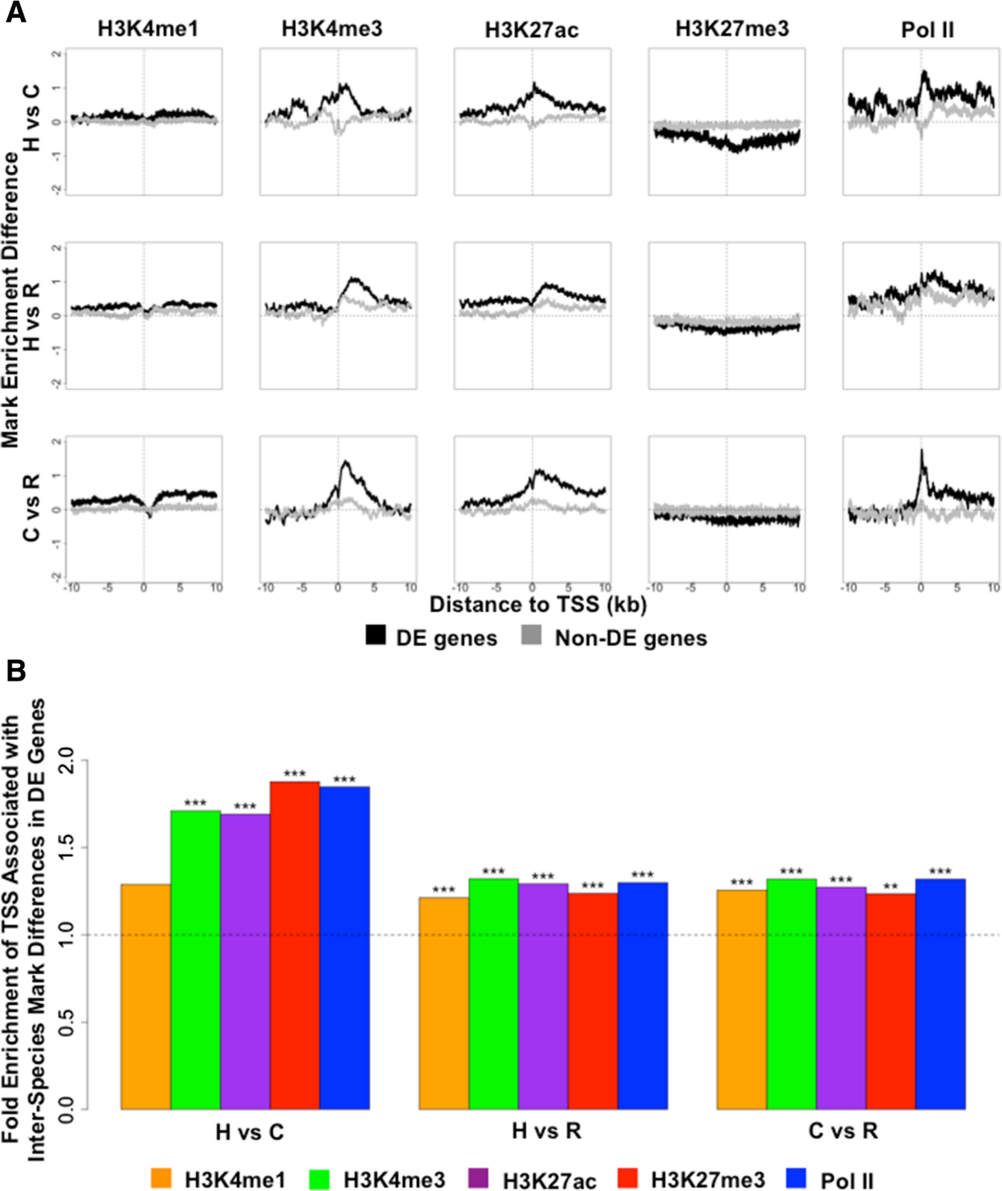
**Differentially expressed genes associate with inter-species differences in mark enrichment at transcription start sites. (A)** Enrichment level differences for the five marks around TSSs of DE genes (black) and non-DE genes (grey) for each pair of species. Mark differences are considered with respect to the species associated with the lower gene expression level. DE genes are determined based on an FDR cutoff of 5%. **(B)** TSS regions associated with inter-species differences in any mark are enriched for DE genes. Plotted is the fold enrichment of TSS regions associated with inter-species differences in enriched marks in DE genes across pairs of species, for genes where the mark enrichment levels and the gene expression levels differ in the expected direction (that is, opposite for H3K27me3, same for the other four marks). Both the TSS regions associated with inter-species differences in enriched marks and DE genes are determined based on an FDR cutoff of 5%. Asterisks indicate significance levels from binomial tests (*P < 0.05, **P < 0.01, ***P < 0.001). C, chimpanzee; H, human; R, rhesus macaque.

We found that DE genes, compared with non-DE genes, are more likely to show inter-species differences in mark enrichment at the TSSs (Figures 3A). The directions of the associations are consistent with our expectations (namely, we observed increased gene expression associated with decrease in H3K27me3 and increase in the other marks and Pol II). In addition, for those genes where the mark enrichment levels and the gene expression levels differ in the expected direction between species (that is, opposite direction for H3K27me3, same direction for the other four marks), DE genes are generally more often associated with inter-species differences in mark enrichment at their TSS regions than expected by chance alone (Figure 3B). These observations are robust with respect to the choice of false discovery rate (FDR) cutoff for classifying DE genes (Additional file 15).

The association of inter-species DE genes and differences in mark enrichment in the corresponding TSS regions across species encouraged us to further explore this relationship. We performed analyses similar to those described above, except that we focused on differences in gene expression level and mark enrichment level between pairs of species.

**Figure 4:**
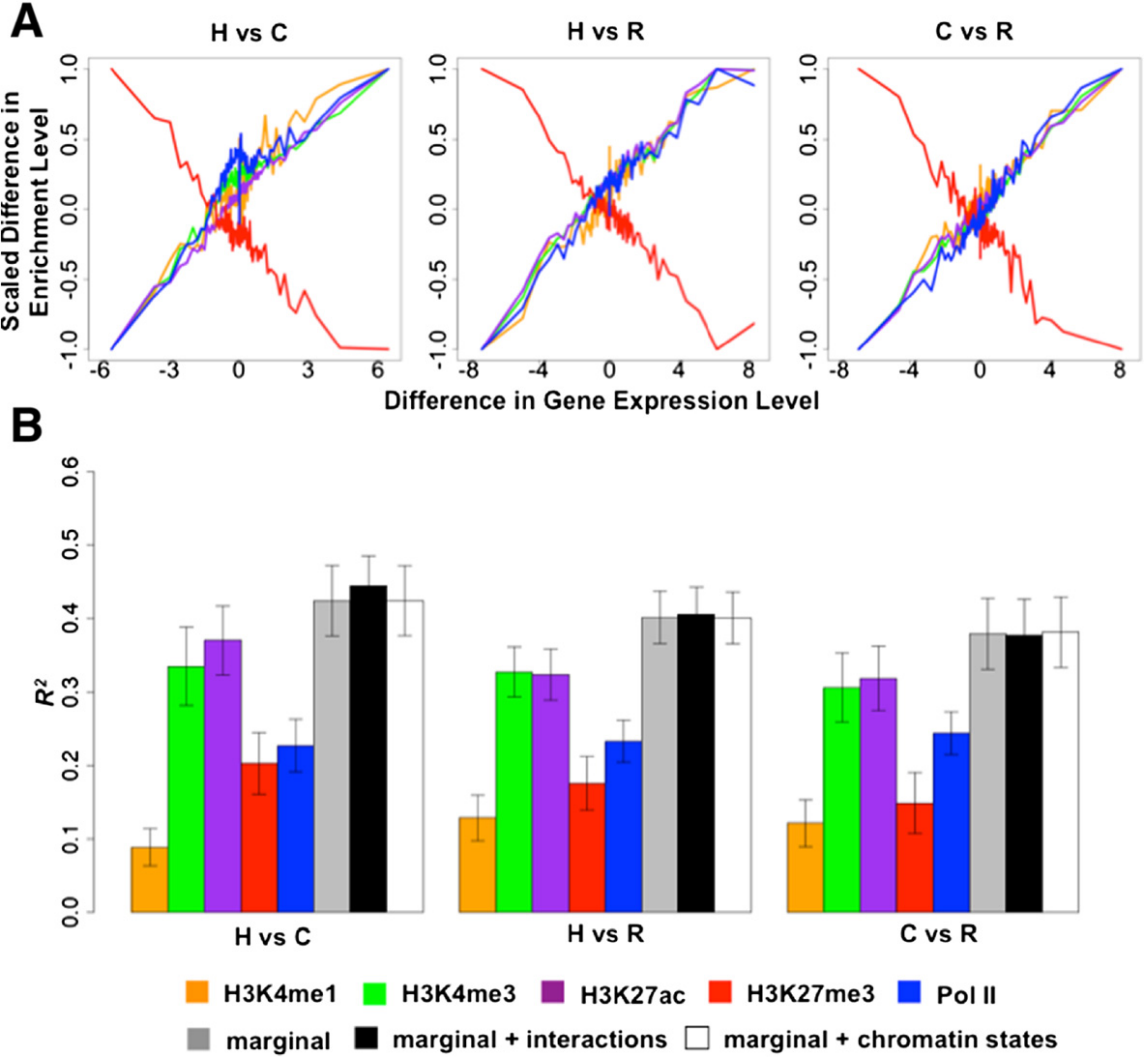
**Differences in mark enrichment level correlate with differences in gene expression level between pairs of primates. (A)** Differences in mark enrichment level is plotted against differences in gene expression level for sliding windows of genes (n = 200) ordered based on the differential expression effect size, for all genes. Differences in enrichment level were obtained in ±2 kb regions near TSSs and scaled to be between -1 and 1. All values are averaged across individuals and across genes in the window. **(B)** Proportion of variance in gene expression level differences explained (R squared) by mark enrichment level differences, for all pairwise comparisons among the three primates. Different linear models are fitted to account for individual marginal effects (five colored bars), combined marginal effects (grey bars), all first-order interaction effects in addition to marginal effects (black bars), and all chromatin state-specific effects in addition to marginal effects (white bars) of the five marks. The DE genes are determined based on an FDR cutoff of 5%. Enrichment level differences are obtained in ±2 kb regions. Error bars indicate standard deviation calculated across 20 split replicates. C, chimpanzee; H, human; R, rhesus macaque.

Considering data from each pair of species at a time (for example, human and chimpanzee), we divided genes into 200-gene groups based on inter-species expression level difference and plotted the mean mark enrichment level differences against the mean gene expression level differences across the species (Figure 4A). We found that differences in mark enrichment level correlate with differences in gene expression level between primates. In particular, the difference in H3K27me3 enrichment level is negatively correlated with gene expression level differences between species, and the enrichment level differences of the other four marks are positively correlated with inter-species differential expression. A few representative patterns are shown in Additional file 16. These observations are robust with respect to the chosen size of the TSS regions (Additional file 17).

To quantitatively measure the proportion of variance in inter-species gene expression level differences explained by the five marks, either individually or combined, we again used a 10-fold cross-validation strategy and applied linear models to calculate R squared in DE genes (Figure 4B; Additional files 18 and 19). We focused on the ±2 kb regions near TSSs as we found these to be most predictive in the analysis of data within species. Each of the five marks explained an appreciable proportion of variance in gene expression level differences between any pairs of species (Figure 4B). The relative importance of the five marks is consistent with that observed within species (Figures 2C and 4B). Together, the five marks explain (in a statistical sense) approximately 40% of the variance in LCL gene expression levels across species (42% between human and chimpanzee, 40% between human and rhesus macaque, and 38% between chimpanzee and rhesus macaque; FDR <5%).

**Figure 5:**
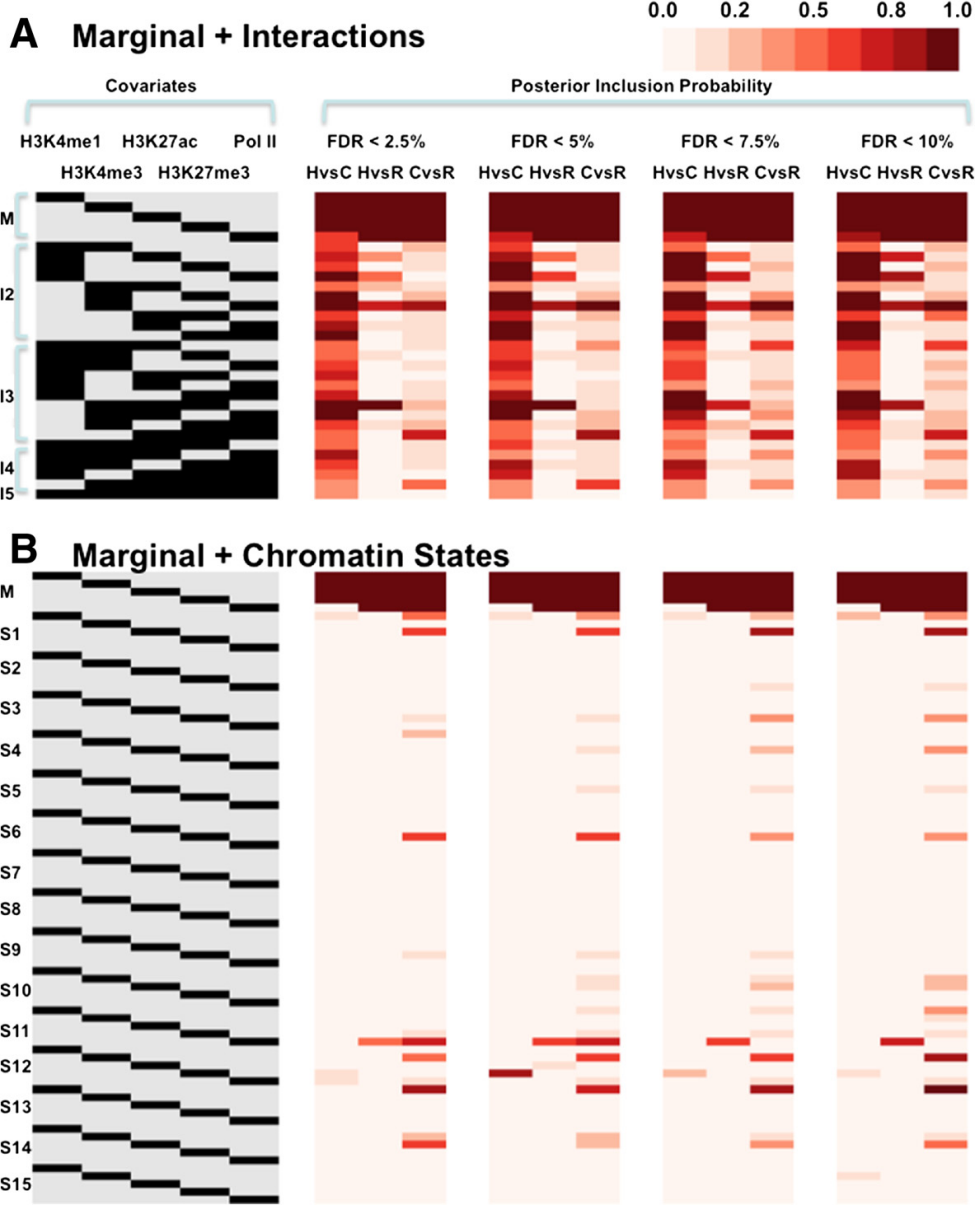
**Importance of marginal and interaction effects from five marks, and their enrichment in different chromatin states, for explaining gene expression level differences between primates. (A)** The left panel lists marginal (M) or interaction terms (I2 to I5) among the five marks, where each row represents an interaction term and each column represents the presence (black) or absence (grey) of a particular mark effect for that interaction term. For example, the first row represents the marginal effect of H3K4me1, and the sixth row represents the interaction effect between H3K4me1 and H3K4me3. The right panel lists the corresponding PIP of each term between any pairs of primates for DE genes classified with different FDR cutoffs. **(B)** The left panel lists marginal (M) or chromatin state-specific terms for 15 chromatin states (S1 to S15) near TSSs, where each column represents the presence (black) or absence (grey) of a particular mark effect for that term. For example, the sixth row represents the state-specific effect of H3K4me1 in chromatin state S1. The right panel lists the corresponding PIP. The PIP measures the importance of each interaction term with higher values indicating higher significance. Mark enrichment level differences and mark enrichment level differences inside chromatin states within ±2 kb regions near TSSs were used for fitting. C, chimpanzee; H, human; R, rhesus macaque. M, marginal effects; I2, interaction term between pairs of marks; I3, interaction term among three marks; I4, interaction term among four marks; I5, interaction term among five marks; S1, active promoter; S2, weak promoter; S3, poised promoter; S4, strong enhancer; S5, strong enhancer; S6, weak enhancer; S7, weak enhancer; S8, insulator; S9, transcription transition; S10, transcription elongation; S11, weak transcription; S12, repressed; S13, heterochroma/lo; S14, repetitive/copy number variation; S15, repetitive/copy number variation.

Finally, we used BVSR to select important marginal and first-order interaction features (Figures 4B and 5A; Additional file 18). Again, we found that all marginal effects are important features that are consistently selected by the model (PIP >0.9 for all FDR cutoffs; Figure 5A). However, only the H3K4me3-Pol II term is consistently selected as an important feature for pairs of species across a range of FDR cutoffs. In addition, modeling the interaction features in addition to the marginal effects does not increase the overall explained variance in gene expression level differences between primates (Figure 4B; Additional file 18).

Finally, we again used BVSR to select important state-specific mark effects with respect to the 15 different chromatin states near TSSs (Figures 4B and 5B; Additional file 18). We found all marginal effects, except for Pol II (which still shows strong evidence in two of the three comparisons), to be consistently selected by the model (Figure 5B). None of the state-specific mark effects in different chromatin states are selected in addition to the marginal effects. Moreover, chromatin states do not contribute much to the variance in gene expression level differences between species, in addition to their marginal effects (Figure 4B; Additional file 18).

## Discussion

### Correlation and causality

As we briefly mention in the results section, it is important to clarify that we use the words 'contribute' and 'explain' to mean a purely statistical conditional relationship between the mark abundance and gene expression levels.

Previous work that focused on molecular mechanisms indicates that variation in Pol II and histone modifications directly affect gene regulation. Specifically, it is well established that Pol II directly transcribes mRNA [52]. It has been shown that H3K4me3 recruits chromatin-remodeling complexes to increase the accessibility of the chromatin to transcriptional machinery and therefore promote gene expression [44,45,65]. It is also generally believed that the other three histone modifications (H3K4me1, H3K27ac, H3K27me3) act in a similar fashion to H3K4me3 to either promote or inhibit gene expression by regulating chromatin accessibility [31]. In particular, the clearance of H3K4me1 is shown to be necessary for the subsequent binding of some transcription factors [66].

On the other hand, recent work (from our lab as well) indicates that oftentimes differences in histone marks are mediated by changes in transcription factor binding [67-69]. Transcription factor binding may be the principle determinant of chromatin state, which is then stabilized or marked by histone modifications. In that sense, the association between changes in histone modification across species and variation in gene expression levels may not indicate a direct causal relationship, but rather an indirect one, possibly mediated by inter-species differences in transcription factor binding.

Indeed, we did not perform experiments here that allow us to directly infer causality. The well-established links from previous studies imply that the quantitative relationship between mark abundance and gene expression level likely reflect, at least in part, a (direct or indirect) causal contribution. In particular, the larger R squared by H3K4me3, H3K27ac and Pol II compared with the other two marks is consistent with the key functions of the three in promoting transcription [44,45,51,65,70]. To better learn the statistical relationship among the marks and gene expression levels, we constructed Bayesian networks using the data in the present study. Interestingly, both within species and between species, only H3K27ac, H3K27me3, and Pol II send directed edges towards RNA, suggesting that the effects from H3K4me1 and H3K4me3 are mediated through the three marks. In addition, both H3K27me3 and Pol II are the critical nodes that receive most input/edges from the other marks (Additional file 20). However, though the Bayesian network is sometimes referred to as the causal network, it only describes the statistical dependency rather than causal relationship among the covariates; the statistical dependency between two covariates could still result from an indirect relationship mediated by unmeasured factors, or induced by some common unmeasured confounding factors.

Therefore, we caution against the over-interpretation of these association results and Bayesian networks, and defer the interrogation of both the direct and directional effects of epigenetic marks on gene expression levels to future studies. It is also possible that other molecular mechanisms are responsible for the correlation and dependency between mark abundance and gene expression levels, at least for a subset of the marks and in a subset of the genes. For example, in some cases a true causal factor may independently affect both gene expression level and histone modifications at the same location (this has been demonstrated previously in other contexts [71,72]), causing correlations or dependency between the two. Our study was not designed to distinguish between all of these possible scenarios.

Regardless of whether the abundance of the four histone modifications and Pol II are truly causally related to variation in gene expression levels, they are only involved in some of the many intermediate steps that a complex machinery takes to convert genome sequence variation, including both *cis*- and *trans*-acting sequence differences, into gene expression variation. The amount of gene expression variation explained by the five marks, therefore, still reflects, at best, only part of the causal contribution of the sequence variation to gene expression variation through transcriptional processes (as opposed to other aspects of the mRNA life cycle, such as decay, splicing and polyadenylation). If the abundance levels of the four histone modifications and Pol II are indeed causal, then the proportion of variance in gene expression levels tracing back to the sequence variation through the five marks is likely smaller than what we have observed here (because the mark abundance variation is at a later step than the sequence variation). If the abundance levels of the five marks are not causal but are by-products of some true causal factors (such as variation in transcription factor binding), then the proportion of variance in gene expression levels tracing back to the sequence variation through these true causal factors could be larger than what we have observed here (because the mark abundance levels are noisy measurements of these causal factors). Moreover, the effects from the sequence variation could be in complicated forms, because simple measurements of sequence conservation and sequence divergence do not predict gene expression level difference between species (Additional file 21). It will be of great interest to reveal the detailed steps of this process and the ultimate contribution of sequence variation to gene expression variation by mapping all the different regulatory checkpoints.

### The chain of events

In our work, we followed the example of previous studies [27,58] and treated the abundance of Pol II and histone modifications equivalently in investigating their relationship to gene expression level variation. We note that numerous studies have established a direct role of Pol II in transcription initiation while pointing to indirect roles of the four histone modifications in transcription initiation through Pol II [31,44,45,51,52,65]. These observations suggest that it might make sense to apply a two-stage analysis to the data. First, we might investigate the contribution of the four histone modifications to Pol II abundance (Figure S20A,C in Additional file 22), and then investigate the contribution of Pol II abundance to gene expression levels (Figures 2C and 4B). However, such naïve analyses ignore the contribution of the four histone modifications to gene expression levels through mechanisms other than regulating the recruitment of Pol II and its abundance levels. For example, studies have shown that Pol II abundance itself is not the sole determinant of transcription initiation, and Pol II can remain in a pausing state without initiating active transcription [73-76]. Such a pausing state can be predicted by histone modifications [70]. Indeed, the constructed Bayesian networks revealed directed effects from H3K27ac and H3K27me3 to gene expression, bypassing Pol II (Additional file 22). In the present study, we also show that modeling the five marks together explains a higher proportion of variation in gene expression level than would be explained by Pol II alone (Figures 2C and 4B). In fact, for both within-species and inter-species analysis, the R squared by the four histone modifications is only slightly smaller than that by the four histone modifications and Pol II (Figure S20B,D in Additional file 22). In addition, the PIPs for each interaction term among the four histone modifications are not sensitive to whether Pol II is included in the analysis or not (that is, the PIPs for each interaction term analyzed without Pol II are similar to those obtained by first analyzing with Pol II but then marginalizing out Pol II; data not shown). As a result of these considerations, we chose to treat the abundance of Pol II and histone modifications equivalently in our study.

### The contribution of interactions between marks

In addition to the marginal effects of the five marks, we also explored the importance of all first-order interaction effects among them. In particular, we identified several notable interaction effects that are important to explaining (in a statistical sense) gene expression level variation within species. Many of these effects are present in important chromatin states identified by other computational methods [33,58]. Two of these interactions, one between H3K4me1 and H3K27ac, and the other between H3K4me1 and H3K27me3, have been recognized to be part of important classes of genomic elements during early development in humans [77]. In addition, we also explored the importance of chromatin states in explaining gene expression variation. We found that H3K4me1 and H3K27ac levels in strong enhancer regions are important to explaining variation in expression level, and both marks have previously shown enrichment in enhancers. However, we found it surprising that the explained proportion of variance in gene expression levels (within or between species) remains largely similar, whether or not we consider all first-order interactions, or whether or not we consider all state-specific mark effects in 15 chromatin states, in addition to the marginal effects in the model. Our results imply that the marginal effects of the five marks dominate the contribution; interaction effects and chromatin state-specific mark effects contribute only a small proportion.

It is possible that we are underpowered to identify important interactions and/or chromatin-specific mark effects. Indeed, measurement noise for any interaction effect is likely the multiplication of noise levels accompanying each marginal effect, and in the case of the inter-species analysis, the sample size is small (because we focused on differentially expressed genes). Additionally, computational models in identifying chromatin states and annotation of TSSs may not be accurate. The statistical challenges notwithstanding, the lack of important and consistent interaction effects as well as chromatin state-specific mark effects in our data is nevertheless an intriguing observation.

### Using lymphoblastoid cell lines as a model system

In the present study, we chose to work with LCLs because they provide abundant material and represent a homogenous cell type from all three species. We note that using LCLs has been criticized previously for two main reasons: that LCLs are cultured cells instead of a primary tissue and are susceptible to batch effects [78,79], and that LCLs require an initial virus transformation that may causes artifacts [80-82]. However, numerous previous studies have demonstrated the usefulness of LCLs in genomics studies [83-92], and have shown that the regulatory architectures identified in LCLs are highly replicable in primary tissues [93-97]. In particular, it has been shown that the patterns of inter-species gene expression level differences in LCLs highly resemble those in primary tissues between primates [98]. In the present study, we also found that the contribution of the five marks to gene expression level variation within species highly resembles those obtained in other tissues or organisms [27,32,60], suggesting that a similar quantitative relationship between the five marks and gene expression level variation exists across multiple species and tissues. In addition, the number of DE genes detected from LCLs in the present study is similar to that obtained from liver tissue in a different study [16], and an average of 28% of the DE genes from our study are also identified as DE genes in theirs (20% between human and chimpanzee, 33% between human and rhesus macaque, and 31% between chimpanzee and rhesus macaque; FDR <5%). Furthermore, the DE genes (human versus chimpanzee and human versus rhesus macaque) detected in the present study are enriched with cerebellum human lineage-specific genes found with a different method in a previous study [99] (53% more than expected; Fisher’s exact test *P*-value = 9.8 × 10^-6^), suggesting their functional relevance in human brain evolution. Therefore, although we acknowledge the potential pitfalls of using LCLs, we believe that they provide a useful and reasonable system, and that the genomic mechanisms we interrogated in LCLs are likely representative of those in primary tissues.

### Final remarks

Even if we assume direct or indirect causality, we note that Pol II and all four histone modifications together do not explain all intra- or inter-species gene expression level variation. Indeed, even with an overly simplified model that accounts for noise in mark enrichment measurement or gene expression measurement (see Materials and methods for details), the 'maximal contribution' from the five marks together to gene expression variation is still estimated to be only 59% within species (60% for human, 59% for chimpanzee, and 58% for rhesus macaque), and 43% for DE genes between species (47% between human and chimpanzee, 43% between human and rhesus macaque, and 40% between chimpanzee and rhesus macaque; FDR <5%). It is likely that other molecular mechanisms (for example, those affecting transcription initiation, mRNA decay, splicing, polyadenylation, and microRNA regulation [100-102]) account for the remaining portion of variation in gene expression levels. We hope that, by collecting comparative genomic data on additional epigenetic and genetic regulatory mechanisms, obtaining more accurate measurements and furthering our analysis on various interactions in the future, we could eventually obtain a better understanding of the detailed molecular mechanisms underlying the evolution of gene expression levels in primates.

## Conclusions

We have explored the extent to which inter-species differences in Pol II and four histone modifications are associated with differences in gene expression levels across primates. We found that all five marks combined explain 40% of the variation in LCL gene expression levels between pairs of species (when we focused on DE genes), which is 5% more than the single most informative mark. These observations suggest that epigenetic modifications are substantially associated with changes in gene expression level among primates and may represent important molecular mechanisms in primate evolution.

## Materials and methods

### Samples and cell culture

Eight LCLs each from human, chimpanzee, and rhesus macaque individuals were obtained from Coriell Institute [103], New Iberia Research Center (University of Louisiana at Lafayette), and New England Primate Research Center (NEPRC, Harvard Medical School). In addition, one input sample from each of the three species was used as control. Cell lines were grown at 37°C in RPMI media with 15% fetal bovine serum, supplemented with 2 mM L-glutamate, 100 IU/ml penicillin, and 100 μg/ml streptomycin.

### ChIPseq and RNAseq

ChIP was performed largely as previously described [25]. In addition to the data collected in this study, we incorporated data from six H3K4me3 ChIP assays performed in one previous study [25] and five Pol II ChIP assays performed in another [101]. For newer samples that were not described in these two previous studies, chromatin was sheared with a Covaris S2 (settings: 40 minutes, duty cycle 20%, intensity 8, 200 cycles/burst, 500 μl at a time in 12 × 24 mm tubes). The amount of antibody used for each ChIP was separately optimized for H3K4me3 (4 μg; Abcam ab8580, Cambridge, MA, USA), H3K4me1 (12 μg; Millipore 07-436, Billerica, MA, USA), H3K27ac (4 μg; Abcam ab4729), H3K27me3 (4 μg; Millipore 07-449), and Pol II (10 μg; Santa Cruz sc-9001, Dallas, TX, USA). Some of the data for the human samples is also used in another study [69].

The quality of each immunoprecipitation was assessed by RT-PCR of positive and negative control genomic regions previously shown to be enriched or not enriched in ENCODE LCL ChIP data for each feature [104]. Successful ChIP assays showed enrichment at the positive control regions relative to the negative control regions in the immunoprecipitated sample compared with the input whole-cell extract from the same individual. We prepared Illumina sequencing libraries from the DNA from each ChIP sample, and from a pooled input sample from each species (containing equal amounts of DNA by mass from each individual in a species) as previously described [105], starting with 20 μl of ChIP output or 4 ng pooled input sample.

Libraries were sequenced in one or more lanes on an Illumina sequencing system using standard Illumina protocols. H3K4me1, H3K4me3, H3K27ac, and H3K27me3 samples were sequenced on a Genome Analyzer II (GAII) system (single end, 36 bp), and Pol II and input samples were sequenced on a HiSeq system (single end, 28 bp and 50 bp, respectively). Input reads were trimmed to 28 bp and 36 bp, where appropriate, for comparison with the reads generated from ChIP samples.

For RNAseq, RNA was extracted and processed to create Illumina sequencing libraries as previously described [25,105]. Each sample was sequenced on one or more lanes of an Illumina GAII system.

### Reads alignment

All sequenced reads were aligned to human (hg19, February 2009), chimpanzee (panTro3, October 2010), or rhesus macaque (rheMac2, January 2006) genome builds with BWA [53] version 0.5.9. Each genome was slightly modified to exclude the Y chromosome, mitochondrial DNA, and regions labeled as random.

We excluded ChIPseq and input reads that were assigned a quality score less than 10, contain more than 2 mismatches or any gaps compared with the reference genome, or are duplicates. We excluded RNAseq reads that were assigned a quality score less than 10 or contain more than 2 mismatches or any gaps relative to the reference genome.

### Classifying genomic regions as enriched

MACS version 1.4.1 [55] was used to identify sharp peaks of enrichment for H3K4me1, H3K4me3, H3K27ac, and Pol II; RSEG version 0.4.4 [54] was used to classify enrichment of broad genomic regions of enrichment for H3K27me3. For MACS, we specified an initial *P*-value threshold that was optimized for each feature (H3K4me1, 0.01; H3K4me3, 0.0001; H3K27ac, 0.001; and Pol II, 0.001), with the appropriate species’ input control file for comparison. Because the chimpanzee sequenced input sample yielded roughly twice the number of reads as the other input samples, to avoid any species bias related to number of input reads, we subsampled the chimpanzee input data to a final number of 40 million reads, which is now comparable to the human and rhesus macaque input samples. For RSEG, we used the 'rseg-diff' function with input control data, with the recommended 20 maximum iterations for hidden Markov model training.

Enriched regions or peaks identified by MACS or RSEG were next filtered to exclude regions or peaks that could not be mapped uniquely in all three primate genomes. To do so, we first divided the genome into 200 bp windows, and we retained those windows that could be mapped to all three primate genomes with gaps less than 100 bp using liftOver [57], and that have at least 80% of bases mappable across all three species (where mappability was measured by the ability of 20 bp sequences to be uniquely mapped to a genome). We then excluded enriched regions or peaks that did not overlap this set of 200 bp windows. To further ensure that regions or peaks of enrichment for features have orthologous positions in human, chimpanzee, and rhesus macaque genomes, we also mapped each region or peak coordinates to the other two genomes with liftOver and excluded enriched regions and peaks that failed to map with at least 20% of the bases aligning to the other genomes.

To minimize the number of falsely identified differences in enrichment status between individuals, we applied two-step cutoffs [25] to classify enriched regions or peaks for each mark. (We chose to present data with this two-step cutoffs procedure because this procedure was also used in other stages of the analysis, though the results presented here are not very sensitive to whether this procedure is applied.) Specifically, for the features analyzed with MACS, we chose a first, stringent FDR cutoff based on the distributions of FDR values associated with identified peaks. A first cutoff of 5% FDR was chosen because we observe a clear enrichment below that value for all features. To select the more relaxed cutoff, we examined the distributions of FDR values for peaks overlapping orthologous positions of peaks that pass the first cutoff (where the orthologous regions were classified by liftOver). These distributions are enriched for small values, which is consistent with individuals of the same or a closely related species having similar epigenetic profiles. We chose secondary FDR cutoffs to capture this enrichment for each feature (H3K4me1, 15%; H3K4me3, 10%; H3K27ac, 15%; and Pol II, 10%).

For H3K27me3, which was analyzed with RSEG, we could not choose cutoffs exactly the same way as described above because RSEG does not produce an FDR value for each enriched region. Instead, for each region classified as enriched, RSEG assigns a domain score, which is the sum of the posterior scores of all bins within the domain. To choose a first, stringent score cutoff, we calculated the proportion of regions classified as enriched by RSEG that overlap regions classified as enriched in ENCODE LCL data [104] at a range of score cutoffs. We chose a first, stringent, score cutoff of 20 because approximately 85% of regions classified as enriched with a score of at least 20 overlapped regions classified as enriched in ENCODE data. To choose a second, more relaxed, score cutoff, we examined all the regions classified as enriched that overlap the orthologous positions of regions classified as enriched by the first cutoff. As expected, over 80% of these regions overlap ENCODE enriched regions, consistent with a low rate of false-positive calls of enrichment among this set of regions. We therefore chose the second, more relaxed cutoff for enrichment to be classification as enriched by RSEG, without a score requirement.

### Mark enrichment level and RNA expression level

We mapped RNA sequencing reads to each orthologous exon, summed values across exons for each gene, and normalized them with respect to the total mapped reads and total exon length to obtain the normalized reads (in RPKM) for each gene. Following convention [27,60,106], we transformed these normalized reads by log2 transformation (after adding a small value to ensure positive values [60,106]), and we termed the resulting value 'gene expression level'. For the five marks, we divided the number of normalized peak reads in different sized regions surrounding the TSSs for each gene by the genome-wide average to obtain mark fold enrichment in these regions. In the case of chromatin state analysis, we retained the peak reads within each given chromatin state, overlapped them with the regions surrounding the TSSs, and normalized for each gene by the genome-wide average. Notice that we did not use the nearest TSS for read assignment because of the potential inaccuracy of TSS annotations. Instead, if a read is close to multiple TSSs then it will be assigned multiple times. We performed square root transformation following previous studies [107], and termed the resulting value 'mark enrichment level', which serves as a measurement of mark abundance. We note that the normalized peak read counts require a step to subtract reads in the corresponding region from input controls, but the final results presented here are not sensitive to whether this step is performed or not.

### Analysis with Bayesian variable selection regression models

BVSR specifies sparse priors on covariates, and has been proven to be effective in selecting important features as well as to be accurate in estimating the proportion of variance in phenotypes explained by all covariates [61,64]. To fit BVSR, we first standardized each covariate to have unit standard deviation. We then used the Markov chain Monte Carlo method (10,000 burn-in iterations and 100,000 sampling iterations) to obtain posterior samples of parameters, using the software GEMMA [64,108,109]. For R squared estimation, we fitted the model in the training set and used the posterior means as coefficient estimates to calculate R squared in the test set. For PIP calculation, we fitted the model using both training and test sets.

### Classifying DE genes and TSS regions associated with inter-species differences in mark enrichment

We tested all genes whose median mark enrichment level or gene expression level across 16 individuals in the species being compared is above zero. To ensure that values are comparable across individuals, we first quantile transformed either the gene expression level or the mark enrichment level across genes in each individual into a standard normal distribution. Afterwards, to guard against model misspecification, for each gene, we further quantile transformed either the gene expression level or the mark enrichment level (in the ±2 kb region near the TSSs) in 16 individuals from the two species being compared into a standard normal distribution. We then fitted a linear model in these individuals with sex as a covariate and species label as a predictor. We tested whether the coefficient for the species label is significantly different from zero. At the same time, we constructed a null distribution by permuting every possible combination of the species label (a total of 6,435 combinations for H3K27ac and 12,870 combinations for the other four marks and RNA), and we calculated the FDR based on this empirical null.

### Overlap between DE genes and TSS regions associated with inter-species differences in mark enrichment

In Figure 3B, for each mark, we focused on genes where the gene expression levels and mark enrichment levels differ between pairs of species in the expected direction. Specifically, for H3K27me3, we focused on genes where the inter-species gene expression level and the mark enrichment level differences are in the opposite direction. For the other four marks, we focused on genes where the inter-species gene expression level and the mark enrichment level differences are in the same direction. Afterwards, we divided the proportion of DE genes that also have TSS regions that are associated with inter-species differences in mark enrichment, by the proportion of non-DE genes that have TSS regions that are associated with inter-species differences in mark enrichment, in order to calculate fold enrichment. We used the binomial test to obtain the corresponding *P*-values.

### Constructing Bayesian networks for five marks and gene expression levels

We used gene expression levels and mark enrichment levels within 2 kb of TSSs to construct Bayesian networks. For each data set, we employed the hill climbing greedy search algorithm to obtain a graph with maximum Bayesian Gaussian score. For interpretation purposes, we encouraged sparsity in the graph by specifying a sparsity-inducing prior on the number of edges (1% prior inclusion probability for each edge in each direction; varying the prior value from 0.1% to 10% does not change the results; in fact, the results are not sensitive to the prior specifications because of the large number of genes used for model fitting). We used the R package bnlearn for model fitting. For biological reasons, we only allowed directed edges from the five marks to RNA but not the other way around. However, even if we do not have this restriction, the graphs learned are largely similar, with the only exception that the RNA-H3K27me3 edge changes direction in rhesus or rhesus-involved comparisons.

### Measuring sequence conservation and difference between species

We used four different measurements for sequence conservation as well as sequence difference between pairs of species in the TSS region. To measure sequence conservation, we obtained the average Phastcons score [110] and the PhyloP score [111,112] in the TSS region. To measure sequence difference, we first used blastn to obtain a list of aligned sequences between pairs of species. We then calculated the proportion of aligned sequence in the TSS region between pairs of species as one measurement, and calculated the average percentage of identity in these aligned sequence in the TSS as another measurement.

### Estimating 'maximal' R squared by accounting for measurement noise

Here, we estimated the 'maximal' R squared by the five marks, by taking into account the measurement noise accompanying both mark enrichment levels and gene expression levels. We considered the following linear model:

$$ {y}_g^o={\displaystyle \sum_{j=1}^5{X}_{gj}^o{\beta}_j+}{\varepsilon}_g,{\varepsilon}_g\sim N\left(0,{\sigma}^2\right), $$

where ${y}_g^o$ is the observed phenotype (that is, gene expression level or gene expression level difference, averaged across individuals) for the *g*th gene, ${x}_{gj}^o$ is the observed *j*th covariate (that is, enrichment level or enrichment level difference for *j*th mark, averaged across individuals) for the *g*th gene, *ε*_*g*_ is the error term, which follows a normal distribution with variance *σ*^2^. For convenience, we assumed that both phenotypes and covariates were already mean centered.

We assumed that both ${y}_g^o$ and ${x}_{gj}^o$ are noisy measurements of the true underlying phenotype *y*_*g*_ and covariate *x*_*gj*_, with the corresponding noises following independent normal distributions:

$$ {y}_g^o={y}_g+{\varepsilon}_g^y,{\varepsilon}_g^y\sim N\left(0,{\sigma}_y^2\right), $$

$$ {x}_{gj}^o={x}_{gj}+{\varepsilon}_g^{xj},{\varepsilon}_g^{xj}\sim N\left(0,{\sigma}_{xj}^2\right), $$

where ${\varepsilon}_g^y$ and ${\varepsilon}_g^{xi}$ are assumed to be independent across genes and independent of each other.

With the above assumptions, we have

$$ E\left({\left({X}^O\right)}^T{X}^O\right)=E\left({X}^TX\right)+G\times D, $$

$$ E\left({\left({X}^O\right)}^T{y}^O\right)=E\left({X}^Ty\right), $$

$$ E\left({\left({y}^O\right)}^T{y}^O\right)=E\left({y}^Ty\right)+G\times {\sigma}_y^2, $$

where *G* is the number of genes, *X*^*o*^ is a *G* by *5* matrix with *gj*th element ${x}_{gj}^o,X$ is a *G* by *5* matrix with *gj*th element *x*_*gj*_, *y*^*o*^ is a *G*-vector with *g*th element ${y}_g^o,y$ is a *G*-vector with *g*th element *y*_*g*_, and $D= diag\left({\sigma}_{x1}^2,{\sigma}_{x2}^2,{\sigma}_{x3}^2,{\sigma}_{x4}^2,{\sigma}_{x5}^2\right)$ is a diagonal matrix.

Therefore, we could approximate the 'maximal' R squared by:

$$ {R}^2=\frac{y^YX{\left({X}^TX\right)}^{-1}{X}^Ty}{y^Yy}\approx \frac{{\left({y}^o\right)}^T{X}^o{\left({\left({X}^o\right)}^T{X}^o-G\times D\right)}^{-1}{\left({X}^o\right)}^T{y}^o}{{\left({y}^o\right)}^T{y}^o-G\times {\sigma}_y^2}, $$

and we replaced ${\sigma}_y^2$ and ${\sigma}_{xi}^2$ with the estimated values:

$$ {\widehat{\sigma}}_y^2=\frac{1}{N^2G}{\displaystyle \sum_{i=1}^N{\displaystyle \sum_{g=1}^G{\left({y}_{ig}^o-{\overline{y}}_g^o\right)}^2,}} $$

$$ {\widehat{\sigma}}_{xj}^2=\frac{1}{N^2G}{\displaystyle \sum_{i=1}^N{\displaystyle \sum_{g=1}^G{\left({x}_{igj}^o-{\overline{x}}_{gj}^o\right)}^2,}} $$

where *N* is the number of individuals.

### Data availability

The data for chimpanzee and rhesus macaque are available in Gene Expression Omnibus (GEO) under accession GSE60269. The data for human were previously deposited under accessions GSE47991 and GSE19480.

## Additional files

Additional file 1: Table S1. Characteristics and sources of lymphoblastoid cell lines. **Table S2.** Number of total sequenced reads for each feature for each individual. **Table S3.** Number of total mapped reads with quality score >10 for each feature for each individual. **Table S4.** Number of sequenced and mapped reads for pooled input samples. **Table S5.** Number of enriched regions/peaks identified for each feature for each individual. **Table S6.** Number of mapped reads in enriched regions/peaks for each mark for each individual. **Table S7.** Number of tested TSS regions and genes. **Table S8.** Number of TSS regions associated with inter-species differences in enriched marks and number of differentially expressed genes identified at different FDR cutoffs. Additional file 2: Figure S1. An illustration of the study design. Additional file 3: Figure S2. Choices of cutoffs for classifying regions as enriched. **(A-H)** Histograms of peaks of H3K4me3 **(A,B)**, H3K4me1 **(C,D)**, H3K27ac (E,F), and Pol II (G,H) enrichment, as classified by MACS, at various FDR thresholds. (A,C,E,G) All peaks with FDR ≤50%; the dashed line indicates the stringent 5% cutoff. (B,D,F,H) Peaks with FDR ≤50% that overlap a peak with FDR ≤5% in another individual; the relaxed FDR cutoff for each feature is marked by a dashed line. **(I,J)** Number of H3K27me3-enriched regions (dark squares, left axis) as classified by RSEG, and the proportion of those regions overlapping ENCODE H3K27me3 peaks (light triangles, right axis) at various score cutoffs up to 200. (I) All enriched regions; the dashed line indicates the stringent 20 score cutoff. (J) Enriched regions that overlap enriched regions with ≥20 score from another individual; the relaxed score cutoff is 0 - that is, any region classified as 'enriched' by RSEG. Additional file 4: Figure S3. Pairwise Spearman’s rank correlations between individuals from the three primates for four histone marks, Pol II, and RNA. Calculations are based on mark abundance in ±2 kb regions near orthologous TSSs for five marks, and on gene expression level in orthologous exons for RNA. C, chimpanzee; H, human; R, rhesus macaque. Additional file 5: Figure S4. Enrichment of Pol II and four histone marks in 15 different chromatin states across the genome in three primates. Error bars indicate standard deviation calculated across individuals. Asterisks indicate significance levels. Additional file 6: Figure S5. Fold enrichment of the five marks in **(A)** ±10 kb and **(B)** ±50 kb regions near TSSs in three primates. Error bars indicate standard deviation calculated across all genes and all individuals. Asterisks indicate significance levels (**P* < 0.05, ***P* < 0.01, ****P* < 0.001). Additional file 7: Figure S6. Pairwise Spearman’s rank correlations between marks in each of the three primates. Calculations are based on mark abundance in **(A)** ±2 kb, **(B)** ±10 kb, and **(C)** ±50 kb regions near orthologous TSSs. Additional file 8: Figure S7. Mark enrichment levels are correlated with gene expression levels in chimpanzee. Legends are identical to those in Figure 2. Additional file 9: Figure S8. Mark enrichment levels are correlated with gene expression levels in rhesus macaque. Legends are identical to those in Figure 2. Additional file 10: Figure S9. Mark enrichment levels are plotted against gene expression levels for sliding windows of genes (n = 200) ordered by increasing expression levels in the three primates. Enrichment levels are obtained in either ±10 kb or ±50 kb regions near TSSs and scaled to be between 0 and 1. All values are averaged across individuals and across genes in the window. Additional file 11: Figure S10. Scatterplot of predicted gene expression levels against true gene expression levels for all analyzed genes in human. Predicted values are obtained based on linear models with either individual marginal effects (colored plots) or all marginal mark effects (grey plot) using mark enrichment levels in ±2 kb regions near TSSs. Additional file 12: Figure S11. Importance of the marginal and first-order interaction effects from the five marks for explaining gene expression levels in the three primates. The left panel lists all interaction terms among the five marks; each row represents an interaction term, and each column represents the presence (black) or absence (grey) of a particular mark effect for that interaction term. For example, the first row represents the marginal effect of Pol II, and the seventh row represents the interaction effect of H3K4me1, H3K4me3, and Pol II. The right panel lists the corresponding posterior inclusion probability of each term in the BVSR in the three species. The posterior inclusion probability measures the importance of each interaction term, with values ranging between 0 and 1; higher values indicate more importance. Mark enrichment levels ±2 kb regions near TSSs are used for fitting. C, chimpanzee; H, human; R, rhesus macaque. Additional file 13: Figure S12. Importance of the mark enrichment in different chromatin states for explaining gene expression levels in the three primates. The left panel lists marginal terms (M) or chromatin state-specific terms for 15 chromatin states (S1 to S15) near TSSs, where each column represents the presence (black) or absence (grey) of a particular mark effect for that term. For example, the first row represents the marginal effect of H3K4me1, and the sixth row represents the effect of H3K4me1 in chromatin state 1 (active promoter) near TSSs. The right panel lists the corresponding posterior inclusion probability of each term in the BVSR in the three species. The posterior inclusion probability measures the importance of each interaction term, with values ranging between 0 and 1; higher values indicate more importance. Mark enrichment levels ±2 kb regions near TSSs are used for fitting. C, chimpanzee; H, human; R, rhesus macaque. M, marginal effects; S1, active promoter; S2, weak promoter; S3, poised promoter; S4, strong enhancer; S5, strong enhancer; S6, weak enhancer; S7, weak enhancer; S8, insulator; S9, transcription transition; S10, transcription elongation; S11, weak transcription; S12, repressed; S13, heterochroma/lo; S14, repetitive/copy number variation; S15, repetitive/copy number variation. Additional file 14: Table S9. List of gene names, peak regions, and their differential expression evidence for pair-wise comparisons (*P*-values and empirical FDRs). Additional file 15: Figure S13. TSS regions associated with inter-species differences in enriched marks are enriched for differentially expressed (DE) genes. TSS regions associated with inter-species differences in enriched marks and DE genes are determined by various FDR cutoffs (2.5%, 7.5%, and 10%). Legends are identical to those in Figure 3. C, chimpanzee; H, human; R, rhesus macaque. Additional file 16: Figure S14. An example of mark abundance and gene expression levels across three species. The x-axis is the distance along a genomic region containing the gene *REX02*. The y-axes show RNAseq reads (black), as well as ChIPseq reads for the five marks (color) and input controls (grey), all scaled with respect to the total mapped read counts. Additional file 17: Figure S15. Differences in mark enrichment level plotted against differences in gene expression level for sliding windows (n = 200) of genes ordered by increasing differences in expression level. Differences in enrichment level are obtained in either ±10 kb or ±50 kb regions near TSSs and scaled to be between -1 and 1. All values are averaged across individuals and across genes in the window. C, chimpanzee; H, human; R, rhesus macaque. Additional file 18: Figure S16. Proportion of variance in gene expression level differences explained (R squared) by mark enrichment level differences, for all pairwise comparisons among the three primates. Different linear models are fitted to account for individual effects (five colored bars), combined marginal effects (grey bars) and all first-order interaction effects in addition to marginal effects (black bars), and all chromatin state-specific effects in addition to marginal effects (white bars) of the five marks. DE genes are determined based on an FDR cutoff of 5%. Enrichment level differences are obtained in ±2 kb regions. Error bars indicate standard deviation calculated across 20 split replicates. C, chimpanzee; H, human; R, rhesus macaque. Additional file 19: Figure S17. Scatterplot of predicted gene expression level differences plotted against true gene expression level differences for DE genes between human and chimpanzee. Predicted values are obtained based on linear models using either individual mark effects (colored plots) or all marginal mark effects (grey plot) with mark enrichment level differences in ±2 kb regions near TSSs. DE genes are determined based on an FDR cutoff of 5%. C, chimpanzee; H, human; R, rhesus macaque. Additional file 20: Figure S18. Bayesian networks describing the statistical dependency among RNA, Pol II, and four histone marks. **(A)** A Bayesian network (left) describes the common statistical dependency among gene expression levels and mark enrichment levels, based on networks inferred from the three species separately (right). **(B)** A Bayesian network (left) describes the common statistical dependency among gene expression differences and enrichment level differences for the five marks, based on networks inferred from the three pair-wise comparisons (right). Additional file 21: Figure S19. Scatterplot of gene expression level differences plotted against sequence conservation and sequence divergence between pairs of species. Two sequence conservation measurements and two sequence divergence measurements are used. DE genes are determined based on an FDR cutoff of 5%. C, chimpanzee; H, human; R, rhesus macaque. Additional file 22: Figure S20. R squared by four histone modifications and Pol II or by four histone modifications alone. **(A)** Proportion of variance in Pol II enrichment level explained by enrichment level of histone modifications. **(B)** Proportion of variance in gene expression level explained by mark enrichment level. **(C)** Proportion of variance in Pol II enrichment level differences explained by enrichment level differences of histone modifications in DE genes. **(D)** Proportion of variance in gene expression level differences explained by mark enrichment level differences in DE genes. Different linear models are fitted to account for combined marginal effects (grey bars) and all first-order interaction effects in addition to marginal effects (black bars). DE genes are determined based on an FDR cutoff of 5%. Enrichment level differences are obtained in ±2 kb regions. Error bars indicate standard deviation calculated across 20 split replicates. C, chimpanzee; H, human; R, rhesus macaque.

## Abbreviations

bp: base pair
BVSR: Bayesian variable selection regression
ChIP: chromatin immunoprecipitation
DE: differentially expressed
FDR: false discovery rate
GAII: Genome Analyzer II
LCL: lymphoblastoid cell line
PCR: polymerase chain reaction
PIP: posterior inclusion probability
Pol II: RNA polymerase II
RPKM: reads per kilobase per million mapped reads
TSS: transcription start site
