# Supplementary material for: Epigenetic modifications are associated with inter-species gene expression variation in primates
Source: Genome Biol. 2014 Dec 3;15:547. doi: 10.1186/s13059-014-0547-3 (PMC4290387; doi:10.1186/s13059-014-0547-3)
Supplement: Additional file 1: Table S1. — Characteristics and sources of lymphoblastoid cell lines. Table S2. Number of total sequenced reads for each feature for each individual. Table S3. Number of total mapped reads with quality score >10 for each feature for each individual. Table S4. Number of sequenced and mapped reads for pooled input samples. Table S5. Number of enriched regions/peaks identified for each feature for each individual. Table S6. Number of mapped reads in enriched regions/peaks for each mark for each individual. Table S7. Number of tested TSS regions and genes. Table S8. Number of TSS regions associated with inter-species differences in enriched marks and number of differentially expressed genes identified at different FDR cutoffs. [file 13059_2014_547_MOESM1_ESM.doc]

**Table S1.** **Characteristics and sources of lymphoblastoid cell lines.**

| **Identifier** | **Species** | **Sex** | **Source** |
| --- | --- | --- | --- |
| GM19239 | *Homo sapiens* | M | Coriell Institute (YRI, NIGMS Human Genetic Cell Repository) |
| GM19141 | *Homo sapiens* | M | Coriell Institute (YRI, NIGMS Human Genetic Cell Repository) |
| GM18505 | *Homo sapiens* | F | Coriell Institute (YRI, NIGMS Human Genetic Cell Repository) |
| GM19238 | *Homo sapiens* | F | Coriell Institute (YRI, NIGMS Human Genetic Cell Repository) |
| GM19193 | *Homo sapiens* | F | Coriell Institute (YRI, NIGMS Human Genetic Cell Repository) |
| GM18508 | *Homo sapiens* | F | Coriell Institute (YRI, NIGMS Human Genetic Cell Repository) |
| GM18507 | *Homo sapiens* | M | Coriell Institute (YRI, NIGMS Human Genetic Cell Repository) |
| GM18522 | *Homo sapiens* | M | Coriell Institute (YRI, NIGMS Human Genetic Cell Repository) |
| S003641 | *Pan troglodytes* | M | Coriell (Yerkes Primates) |
| Min19919 | *Pan troglodytes* | M | University of Louisiana at Lafayette New Iberia Research Center |
| Min19913 | *Pan troglodytes* | M | University of Louisiana at Lafayette New Iberia Research Center |
| S003659 | *Pan troglodytes* | F | Coriell Institute (Yerkes Primates) |
| AG18358 | *Pan troglodytes* | F | Coriell Institute, NIA Cell Repository |
| AG18359 | *Pan troglodytes* | F | Coriell Institute, NIA Cell Repository |
| S003610 | *Pan troglodytes* | M | Coriell Institute (Yerkes Primates) |
| S004973 | *Pan troglodytes* | M | Coriell Institute (Yerkes Primates) |
| Mm 181-96 | *Macaca mulatta* | M | Harvard Medical School, NEPRC |
| Mm 249-97 | *Macaca mulatta* | M | Harvard Medical School, NEPRC |
| Mm 290-96 | *Macaca mulatta* | M | Harvard Medical School, NEPRC |
| Mm 153-99 | *Macaca mulatta* | M | Harvard Medical School, NEPRC |
| Mm 150-99 | *Macaca mulatta* | F | Harvard Medical School, NEPRC |
| Mm 173-02 | *Macaca mulatta* | F | Harvard Medical School, NEPRC |
| Mm 265-95 | *Macaca mulatta* | M | Harvard Medical School, NEPRC |
| Mm 256-95 | *Macaca mulatta* | M | Harvard Medical School, NEPRC |

**Table S2. Number of total sequenced reads for each feature for each individual.**

| **Identifier** | **H3K4me1** | **H3K4me3** | **H3K27ac** | **H3K27me3** | **Pol II** | **RNA** |
| --- | --- | --- | --- | --- | --- | --- |
| GM19239 | 41,487,566 | 41,049,455 | 42,985,514 | 40,014,399 | 204,235,178 | 16,824,406 |
| GM19141 | 35,582,535 | 35,600,431 | 36,602,602 | 41,396,087 | 202,946,440 | 19,405,296 |
| GM18505 | 55,852,093 | 45,099,741 | 41,619,485 | 42,418,783 | 207,829,892 | 15,982,288 |
| GM19238 | 33,585,084 | 31,528,815 | 42,518,152 | 41,259,657 | 409,346,874 | 15,625,031 |
| GM19193 | 33,200,484 | 33,272,920 | 42,554,122 | 42,376,103 | 167,057,685 | 15,710,798 |
| GM19508 | 43,192,419 | 44,379,547 | 42,590,850 | 43,683,243 | 206,275,097 | 17,156,262 |
| GM18507 | 82,589,169 | 42,273,137 | 36,534,335 | 41,882,091 | 207,080,008 | 25,389,574 |
| GM18522 | 29,251,590 | 42,253,530 | 41,753,448 | 41,465,617 | 206,977,123 | 16,767,897 |
| H Average | 44,342,618 | 39,432,197 | 40,894,814 | 41,811,998 | 226,468,537 | 17,857,694 |
|  |  |  |  |  |  |  |
| S003641 | 66,639,859 | 51,034,333 | 41,565,970 | 39,814,932 | 199,846,847 | 21,711,554 |
| Min19919 | 35,618,656 | 47,275,050 | 42,332,278 | 44,308,470 | 185,894,792 | 15,265,605 |
| Min19913 | 57,020,873 | 51,154,147 | 41,994,654 | 42,759,984 | 188,485,158 | 4,519,359 |
| S003659 | 37,697,003 | 39,835,109 | 31,842,862 | 42,331,853 | 209,359,996 | 8,216,282 |
| AG18358 | 34,798,238 | 42,334,865 | 30,551,526 | 42,366,663 | 207,245,396 | 17,332,962 |
| AG18359 | 34,756,174 | 43,437,165 | 42,498,967 | 41,352,348 | 227,500,046 | 8,076,830 |
| S003610 | 75,620,326 | 41,483,139 | 42,021,629 | 41,783,916 | 385,976,580 | 4,356,426 |
| S004973 | 41,568,180 | 35,009,166 | 42,089,691 | 40,349,899 | 197,989,651 | 8,127,615 |
| C Average | 47,964,914 | 43,945,372 | 39,362,197 | 41,883,508 | 225,287,308 | 10,950,829 |
|  |  |  |  |  |  |  |
| Mm 181-96 | 33,258,006 | 47,737,304 | n/a | 42,762,302 | 207,499,540 | 5,794,635 |
| Mm 249-97 | 69,108,961 | 50,796,934 | 41,243,683 | 41,327,611 | 209,493,582 | 14,354,061 |
| Mm 290-96 | 64,245,966 | 51,288,993 | 39,590,650 | 40,543,059 | 222,462,323 | 13,983,869 |
| Mm 153-99 | 36,056,912 | 31,178,848 | 41,872,385 | 37,704,594 | 202,836,916 | 6,935,762 |
| Mm 150-99 | 38,729,216 | 39,225,718 | 35,416,786 | 38,684,767 | 51,710,240 | 13,335,185 |
| Mm 173-02 | 72,813,260 | 41,572,606 | 42,832,258 | 39,205,449 | 210,375,074 | 15,212,293 |
| Mm 265-95 | 39,295,111 | 45,043,323 | 43,603,117 | 40,248,980 | 192,637,781 | 8,722,182 |
| Mm 256-95 | 39,014,122 | 66,872,475 | 38,527,919 | 38,806,015 | 206,845,332 | 8,848,655 |
| R Average | 49,065,194 | 45,843,485 | 39,664,990 | 39,910,347 | 187,982,598 | 10,898,330 |

**Table S3. Number of total mapped reads with quality score >10 for each feature for each individual.**

| **Identifier** | **H3K4me1** | **H3K4me3** | **H3K27ac** | **H3K27me3** | **Pol II** | **RNA** |
| --- | --- | --- | --- | --- | --- | --- |
| GM19239 | 26,017,939 | 26,064,614 | 33,493,274 | 26,176,943 | 56,299,972 | 11,483,161 |
| GM19141 | 10,396,960 | 21,802,862 | 29,819,188 | 28,575,285 | 50,170,381 | 13,275,048 |
| GM18505 | 20,973,695 | 22,899,405 | 32,208,719 | 31,350,656 | 25,639,798 | 10,581,852 |
| GM19238 | 11,995,885 | 18,097,838 | 33,695,704 | 30,843,089 | 76,701,676 | 10,406,792 |
| GM19193 | 19,240,073 | 23,763,462 | 32,919,074 | 31,414,276 | 46,912,132 | 9,693,276 |
| GM18508 | 23,094,712 | 32,960,298 | 33,765,025 | 32,714,493 | 37,357,158 | 11,319,365 |
| GM18507 | 30,054,288 | 27,118,043 | 29,380,811 | 30,125,374 | 26,240,949 | 16,452,612 |
| GM18522 | 16,039,417 | 21,543,352 | 33,025,934 | 26,367,959 | 50,163,104 | 10,904,147 |
| H Average | 19,726,621 | 24,281,234 | 32,288,466 | 29,696,009 | 46,185,646 | 11,764,531 |
|  |  |  |  |  |  |  |
| S003641 | 15,189,103 | 35,249,152 | 31,551,844 | 28,352,938 | 26,252,706 | 9,263,678 |
| Min19919 | 9,971,397 | 34,563,572 | 31,228,738 | 28,854,196 | 20,153,172 | 10,073,252 |
| Min19913 | 30,527,178 | 34,694,785 | 31,113,435 | 27,695,262 | 28,280,526 | 2,847,452 |
| S003659 | 18,685,059 | 20,821,445 | 24,542,085 | 29,478,467 | 15,619,668 | 5,434,537 |
| AG18358 | 20,491,297 | 22,706,890 | 23,436,348 | 30,281,451 | 23,898,662 | 11,439,295 |
| AG18359 | 17,437,745 | 31,058,065 | 31,706,275 | 24,742,497 | 19,324,888 | 5,342,048 |
| S003610 | 22,121,269 | 25,412,098 | 31,352,543 | 25,231,592 | 29,136,386 | 2,896,449 |
| S004973 | 22,028,905 | 25,400,663 | 31,352,495 | 29,308,801 | 15,409,922 | 5,412,334 |
| C Average | 19,556,494 | 28,738,333 | 29,535,470 | 27,993,150 | 22,259,491 | 6,588,630 |
|  |  |  |  |  |  |  |
| Mm 181-96 | 20,399,540 | 30,790,972 | n/a | 30,188,863 | 23,643,031 | 3,759,255 |
| Mm 249-97 | 20,967,823 | 32,593,975 | 29,419,374 | 27,226,446 | 45,333,770 | 9,224,547 |
| Mm 290-96 | 27,554,631 | 34,694,363 | 30,179,458 | 19,728,076 | 29,168,549 | 9,118,418 |
| Mm 153-99 | 10,198,710 | 21,045,305 | 31,780,955 | 24,426,300 | 49,634,508 | 4,221,410 |
| Mm 150-99 | 15,000,444 | 25,407,485 | 27,700,232 | 25,654,399 | 19,641,359 | 8,177,007 |
| Mm 173-02 | 19,649,219 | 29,590,695 | 30,955,725 | 27,868,722 | 31,043,169 | 9,411,944 |
| Mm 265-95 | 23,999,109 | 31,019,746 | 31,639,019 | 29,200,054 | 17,484,876 | 5,151,862 |
| Mm 256-95 | 22,691,535 | 43,838,420 | 30,027,729 | 26,923,497 | 35,110,394 | 5,213,118 |
| R Average | 20,057,626 | 31,122,620 | 28,250,094 | 26,402,045 | 31,382,457 | 6,784,695 |

**Table S4. Number of sequenced and mapped reads for pooled input samples.**

| **Species** | **Length (bp)** | **Sequenced Reads** | **Mapped Reads** |
| --- | --- | --- | --- |
| Human | 28 | 137,567,396 | 40,787,484 |
| Chimpanzee | 28 | 140,643,585 | 91,517,810 |
| Rhesus Macaque | 28 | 118,225,414 | 37,027,106 |
| Human | 36 | 137,567,396 | 43,370,536 |
| Chimpanzee | 36 | 140,643,585 | 97,023,371 |
| Rhesus Macaque | 36 | 118,225,414 | 39,145,138 |

**Table S5. Number of enriched regions/peaks identified for each mark for each individual.**

| **Identifier** | **H3K4me1** | **H3K4me3** | **H3K27ac** | **H3K27me3** | **Pol II** |
| --- | --- | --- | --- | --- | --- |
| GM19239 | 67,491 | 20,942 | 77,300 | 3,725 | 23,524 |
| GM19141 | 472,955 | 21,107 | 89,924 | 2,603 | 33,403 |
| GM18505 | 112,043 | 50,260 | 84,642 | 16,716 | 45,791 |
| GM19238 | 262,300 | 23,069 | 77,664 | 1,663 | 20,942 |
| GM19193 | 135,202 | 20,771 | 65,404 | 1,705 | 30,516 |
| GM18508 | 87,853 | 44,969 | 68,646 | 10,697 | 38,456 |
| GM18507 | 71,764 | 18,949 | 85,867 | 3,029 | 46,087 |
| GM18522 | 130,845 | 20,817 | 80,370 | 2,869 | 45,157 |
| H Average | 167,557 | 27,610 | 78,727 | 5,376 | 35,484 |
|  |  |  |  |  |  |
| S003641 | 255,794 | 50,969 | 87,391 | 4,999 | 170,066 |
| Min19919 | 253,282 | 61,826 | 101,378 | 2,673 | 163,895 |
| Min19913 | 226,995 | 58,854 | 94,307 | 2,977 | 133,164 |
| S003659 | 244,790 | 33,113 | 91,816 | 12,895 | 185,397 |
| AG18358 | 108,170 | 24,476 | 118,514 | 8,349 | 178,568 |
| AG18359 | 181,939 | 63,838 | 88,051 | 2,303 | 276,527 |
| S003610 | 251,632 | 34,052 | 71,110 | 912 | 99,250 |
| S004973 | 201,913 | 54,677 | 96,213 | 16,699 | 123,127 |
| C Average | 215,564 | 47,726 | 93,598 | 6,476 | 166,249 |
|  |  |  |  |  |  |
| Mm 181-96 | 82,827 | 47,699 | n/a | 10,274 | 60,586 |
| Mm 249-97 | 147,789 | 57,451 | 92,414 | 5,361 | 55,669 |
| Mm 290-96 | 100,991 | 54,773 | 81,953 | 1,420 | 48,295 |
| Mm 153-99 | 290,150 | 32,549 | 65,860 | 1,382 | 33,594 |
| Mm 150-99 | 335,598 | 16,714 | 60,110 | 2,435 | 105,675 |
| Mm 173-02 | 143,724 | 52,793 | 78,749 | 11,915 | 33,409 |
| Mm 265-95 | 146,033 | 36,661 | 79,902 | 5,485 | 94,428 |
| Mm 256-95 | 108,888 | 31,402 | 70,679 | 2,710 | 31,353 |
| R Average | 169,500 | 41,255 | 75,667 | 5,123 | 57,876 |

**Table S6. Number of mapped reads in enriched regions/peaks for each mark for each individual.**

| **Identifier** | **H3K4me1** | **H3K4me3** | **H3K27ac** | **H3K27me3** | **Pol II** |
| --- | --- | --- | --- | --- | --- |
| GM19239 | 5,065,287 | 6,939,262 | 12,373,667 | 8,539,096 | 4,340,979 |
| GM19141 | 3,379,461 | 5,256,206 | 8,728,313 | 10,082,096 | 4,860,603 |
| GM18505 | 5,820,762 | 4,686,621 | 10,768,626 | 9,742,980 | 2,178,829 |
| GM19238 | 3,766,385 | 4,488,317 | 9,116,144 | 7,161,920 | 5,443,774 |
| GM19193 | 5,321,616 | 4,525,150 | 5,899,666 | 7,310,984 | 4,022,746 |
| GM18508 | 4,243,844 | 14,399,582 | 9,072,231 | 11,501,092 | 4,267,625 |
| GM18507 | 6,514,791 | 7,565,359 | 8,488,020 | 10,880,787 | 1,526,459 |
| GM18522 | 4,786,421 | 9,278,879 | 10,417,544 | 5,984,255 | 9,000,241 |
| H Average | 4,862,321 | 7,142,422 | 9,358,026 | 8,900,401 | 4,455,157 |
|  |  |  |  |  |  |
| S003641 | 5,104,317 | 8,616,339 | 8,900,744 | 14,901,179 | 3,254,108 |
| Min19919 | 2,711,051 | 10,721,998 | 8,604,092 | 8,021,023 | 3,010,100 |
| Min19913 | 8,785,491 | 8,441,823 | 9,400,862 | 8,031,401 | 3,271,205 |
| S003659 | 5,585,132 | 4,840,756 | 6,267,801 | 7,557,834 | 2,635,452 |
| AG18358 | 4,369,308 | 5,306,297 | 5,736,420 | 5,407,291 | 3,512,369 |
| AG18359 | 3,025,608 | 16,280,890 | 9,677,877 | 4,528,788 | 4,872,072 |
| S003610 | 6,960,849 | 8,067,038 | 16,141,580 | 1,430,542 | 5,336,914 |
| S004973 | 5,928,785 | 9,196,317 | 10,280,294 | 10,659,480 | 1,797,786 |
| C Average | 5,308,818 | 8,933,932 | 9,376,209 | 7,567,192 | 3,461,251 |
|  |  |  |  |  |  |
| Mm 181-96 | 4,774,201 | 5,458,004 | n/a | 12,428,711 | 1,954,373 |
| Mm 249-97 | 5,551,946 | 14,874,774 | 8,209,221 | 11,185,915 | 8,524,714 |
| Mm 290-96 | 6,913,110 | 11,958,029 | 6,310,769 | 2,402,039 | 4,008,854 |
| Mm 153-99 | 3,388,577 | 6,818,286 | 9,339,266 | 4,162,292 | 5,200,122 |
| Mm 150-99 | 4,692,849 | 7,111,106 | 4,860,517 | 7,791,163 | 2,677,656 |
| Mm 173-02 | 6,151,745 | 11,919,638 | 10,377,568 | 9,312,096 | 2,505,067 |
| Mm 265-95 | 7,795,755 | 11,757,890 | 9,952,487 | 14,920,348 | 1,512,686 |
| Mm 256-95 | 6,180,534 | 18,291,720 | 8,534,397 | 9,415,428 | 3,030,117 |
| R Average | 5,681,090 | 11,023,681 | 8,226,318 | 8,952,249 | 3,676,699 |

**Table S7. Number of tested TSS regions and genes**.

|  | **H3K4me1** | **H3K4me3** | **H3K27ac** | **H3K27me3** | **Pol II** | **RNA** |
| --- | --- | --- | --- | --- | --- | --- |
| H vs C | 13492 | 11497 | 11209 | 5458 | 10186 | 13082 |
| H vs R | 13052 | 11354 | 10584 | 5618 | 9678 | 13010 |
| C vs R | 13601 | 11979 | 10828 | 5435 | 10338 | 12615 |

**Table S8. Number of TSS regions associated with interspecies differences in enrichment of marks and number of DE genes identified at different FDR cutoffs.**

|  | **H3K4me1** | **H3K4me3** | **H3K27ac** | **H3K27me3** | **Pol II** | **RNA** |
| --- | --- | --- | --- | --- | --- | --- |
| **FDR<2.5%** |  |  |  |  |  |  |
| H vs C | 0 | 2302 | 2412 | 239 | 1129 | 2999 |
| H vs R | 2484 | 4387 | 4850 | 1056 | 3067 | 5710 |
| C vs R | 2553 | 4238 | 4714 | 677 | 2613 | 5156 |
| **FDR<7.5%** |  |  |  |  |  |  |
| H vs C | 308 | 3540 | 3655 | 631 | 1959 | 4393 |
| H vs R | 3921 | 5772 | 5975 | 1749 | 4211 | 7145 |
| C vs R | 3929 | 5447 | 5972 | 1242 | 3770 | 6464 |
| **FDR<10%** |  |  |  |  |  |  |
| H vs C | 477 | 3953 | 4163 | 799 | 2337 | 4810 |
| H vs R | 4464 | 6190 | 6288 | 1989 | 4548 | 7560 |
| C vs R | 4481 | 5805 | 6339 | 1423 | 4209 | 6867 |
