# Supplementary figures and images for: Epigenetic modifications are associated with inter-species gene expression variation in primates
Source: Genome Biol. 2014 Dec 3;15:547. doi: 10.1186/s13059-014-0547-3 (PMC4290387; doi:10.1186/s13059-014-0547-3)

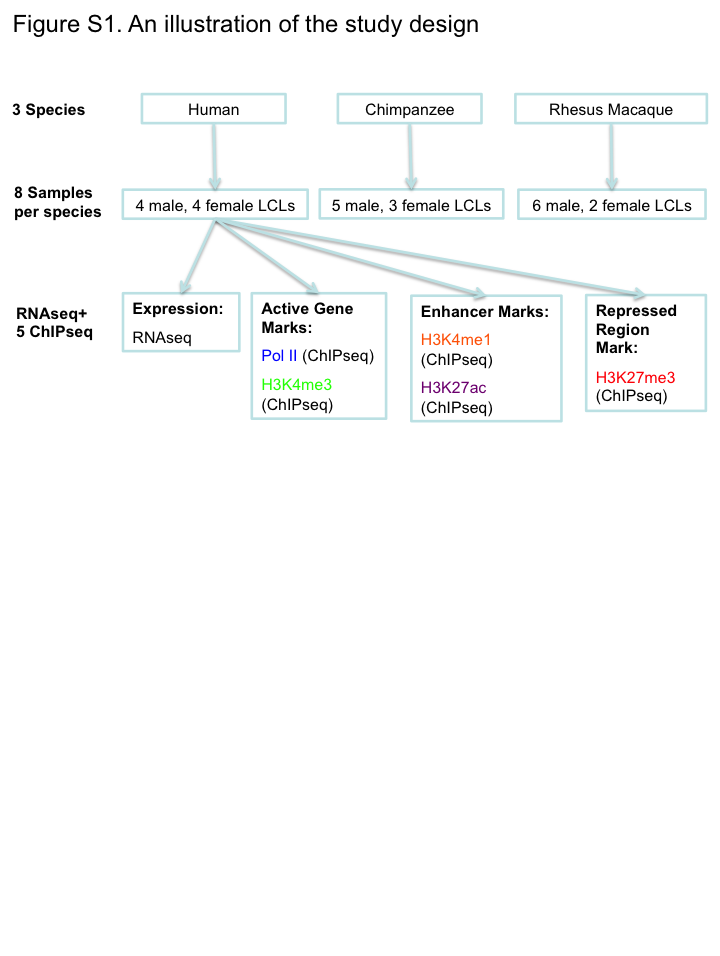

Supplement: Additional file 2: Figure S1. — An illustration of the study design. [file 13059_2014_547_MOESM2_ESM.tiff]

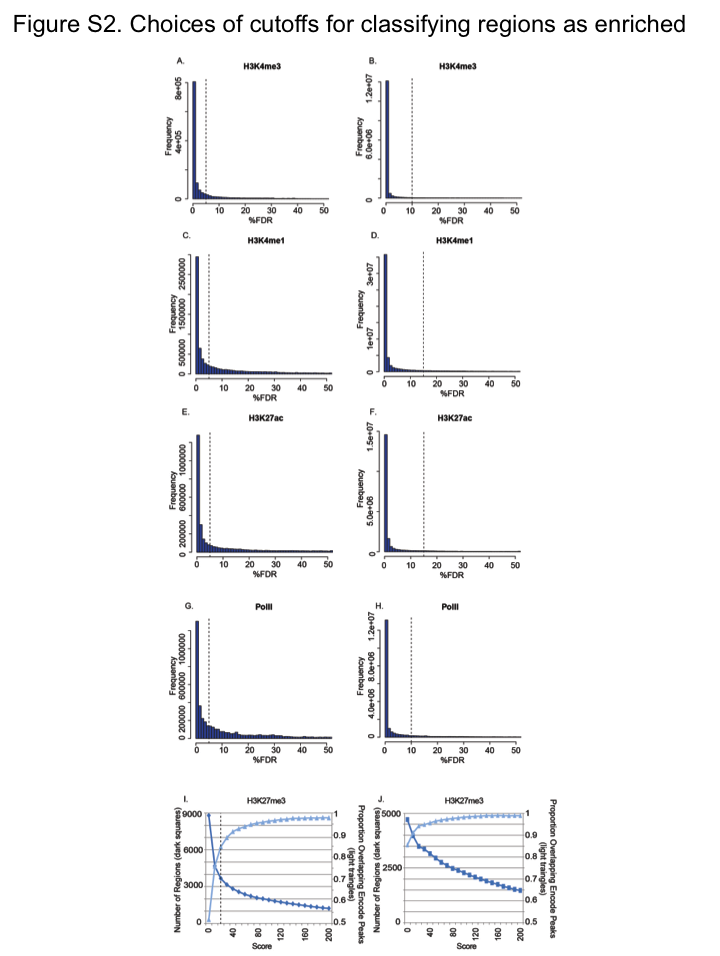

Supplement: Additional file 3: Figure S2. — Choices of cutoffs for classifying regions as enriched. (A-H) Histograms of peaks of H3K4me3 (A,B), H3K4me1 (C,D), H3K27ac (E,F), and Pol II (G,H) enrichment, as classified by MACS, at various FDR thresholds. (A,C,E,G) All peaks with FDR ≤50%; the dashed line indicates the stringent 5% cutoff. (B,D,F,H) Peaks with FDR ≤50% that overlap a peak with FDR ≤5% in another individual; the relaxed FDR cutoff for each feature is marked by a dashed line. (I,J) Number of H3K27me3-enriched regions (dark squares, left axis) as classified by RSEG, and the proportion of those regions overlapping ENCODE H3K27me3 peaks (light triangles, right axis) at various score cutoffs up to 200. (I) All enriched regions; the dashed line indicates the stringent 20 score cutoff. (J) Enriched regions that overlap enriched regions with ≥20 score from another individual; the relaxed score cutoff is 0 - that is, any region classified as 'enriched' by RSEG. [file 13059_2014_547_MOESM3_ESM.tiff]

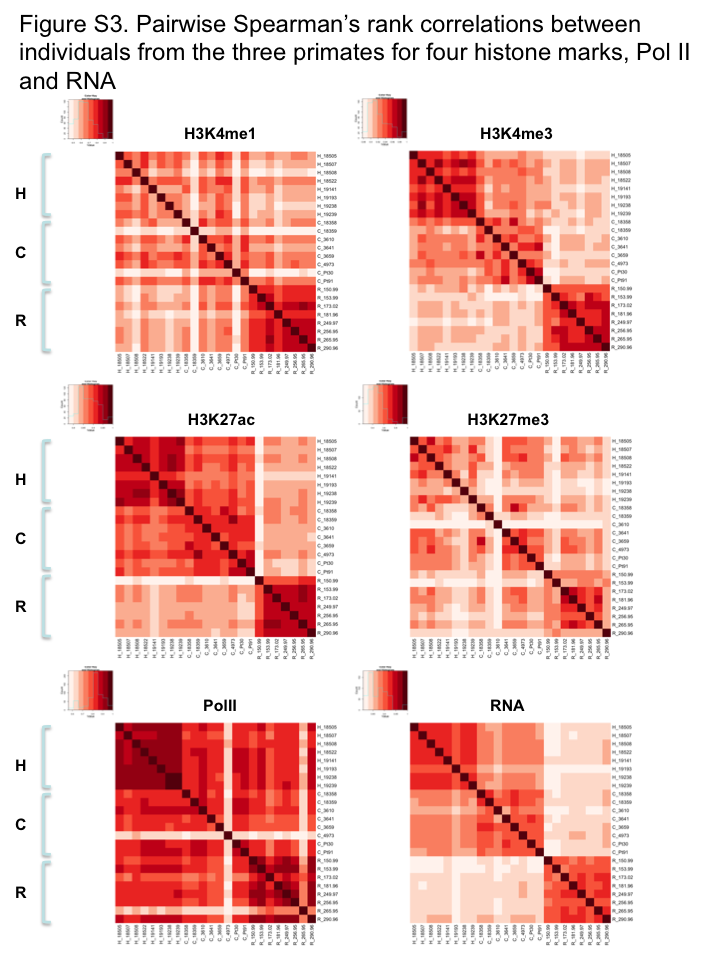

Supplement: Additional file 4: Figure S3. — Pairwise Spearman’s rank correlations between individuals from the three primates for four histone marks, Pol II, and RNA. Calculations are based on mark abundance in ±2 kb regions near orthologous TSSs for five marks, and on gene expression level in orthologous exons for RNA. C, chimpanzee; H, human; R, rhesus macaque. [file 13059_2014_547_MOESM4_ESM.tiff]

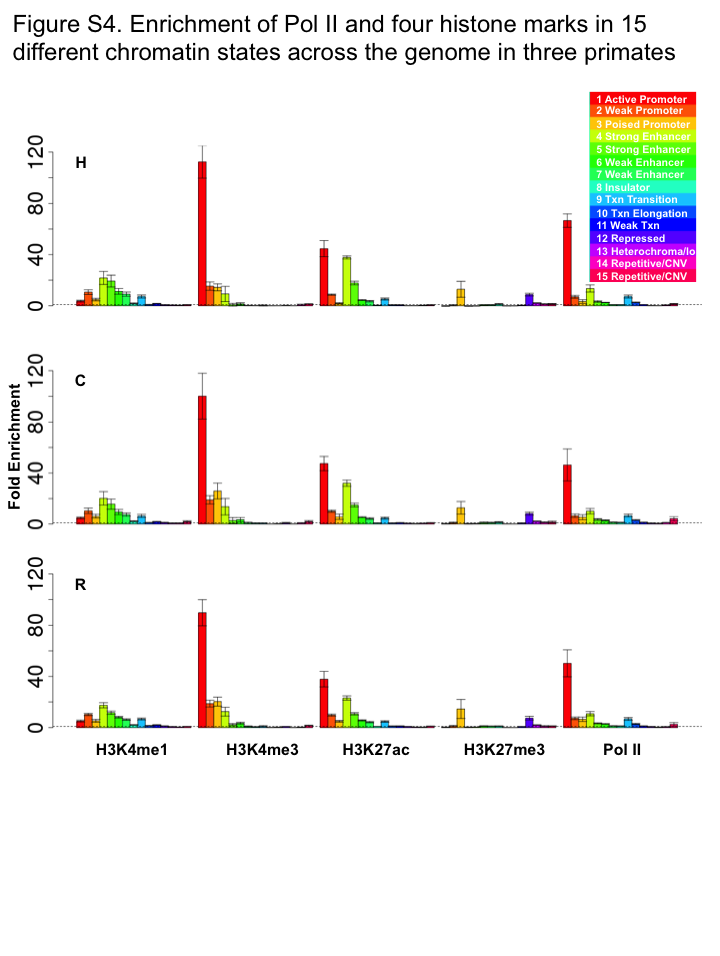

Supplement: Additional file 5: Figure S4. — Enrichment of Pol II and four histone marks in 15 different chromatin states across the genome in three primates. Error bars indicate standard deviation calculated across individuals. Asterisks indicate significance levels. [file 13059_2014_547_MOESM5_ESM.tiff]

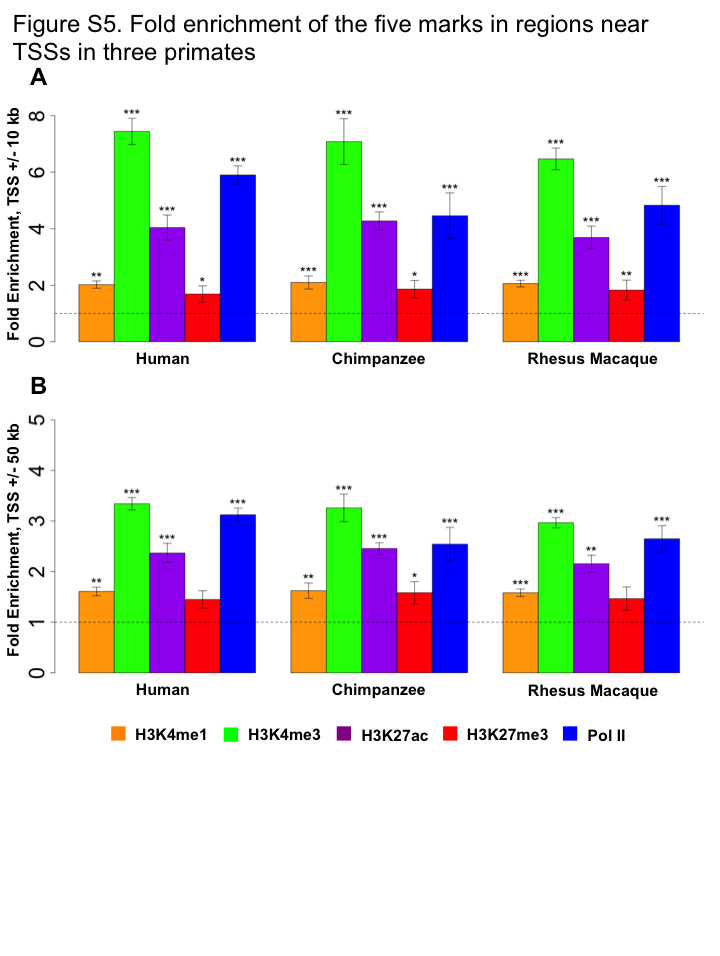

Supplement: Additional file 6: Figure S5. — Fold enrichment of the five marks in (A) ±10 kb and (B) ±50 kb regions near TSSs in three primates. Error bars indicate standard deviation calculated across all genes and all individuals. Asterisks indicate significance levels (*P < 0.05, **P < 0.01, ***P < 0.001). [file 13059_2014_547_MOESM6_ESM.tiff]

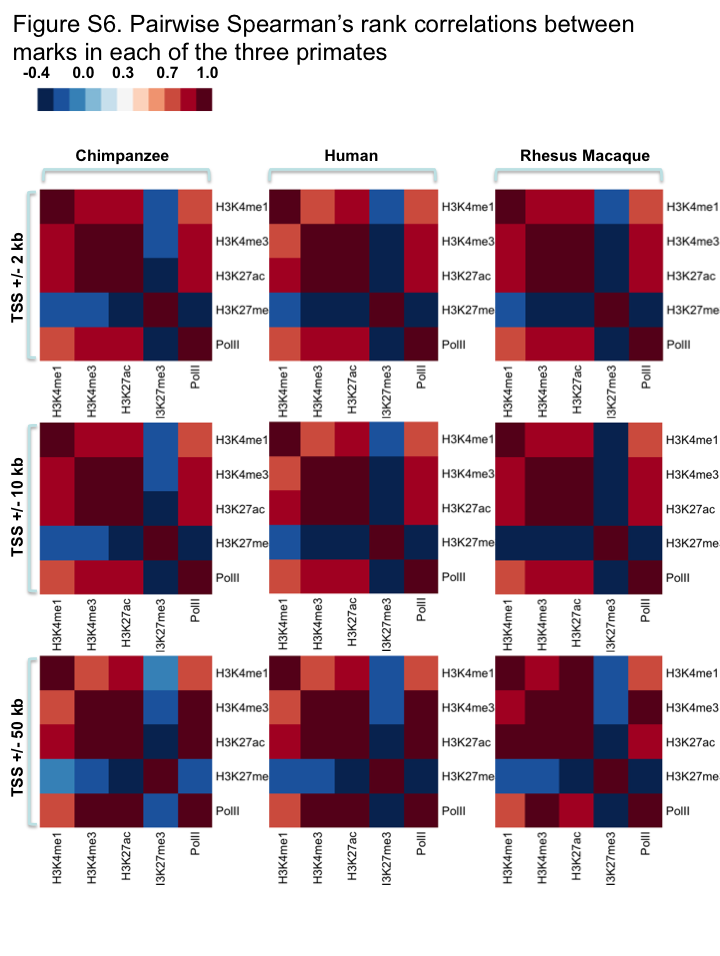

Supplement: Additional file 7: Figure S6. — Pairwise Spearman’s rank correlations between marks in each of the three primates. Calculations are based on mark abundance in (A) ±2 kb, (B) ±10 kb, and (C) ±50 kb regions near orthologous TSSs. [file 13059_2014_547_MOESM7_ESM.tiff]

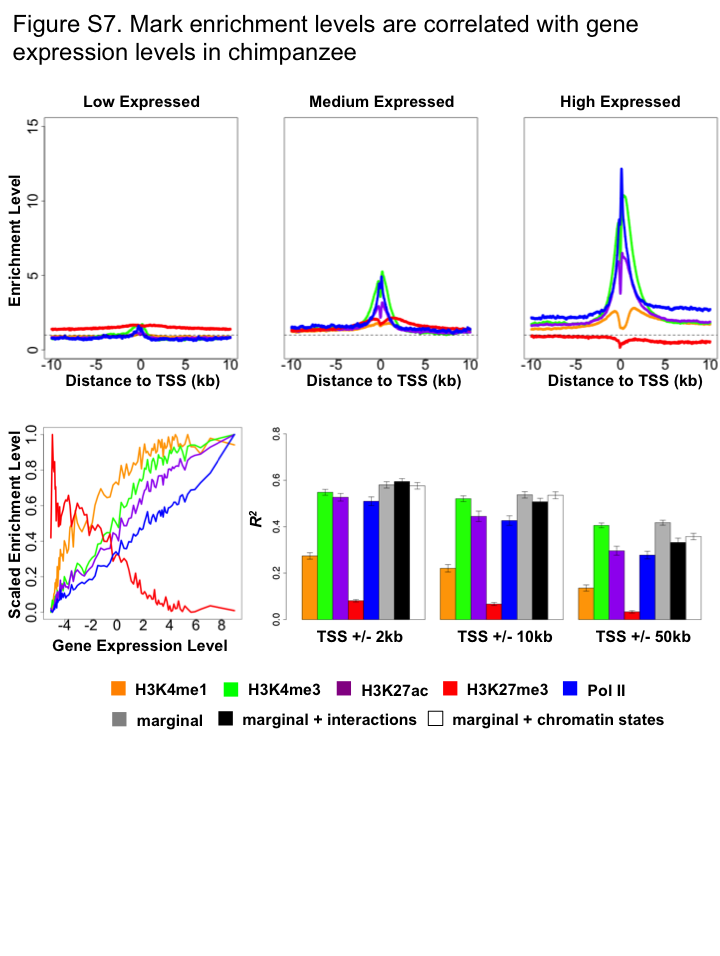

Supplement: Additional file 8: Figure S7. — Mark enrichment levels are correlated with gene expression levels in chimpanzee. Legends are identical to those in Figure 2. [file 13059_2014_547_MOESM8_ESM.tiff]

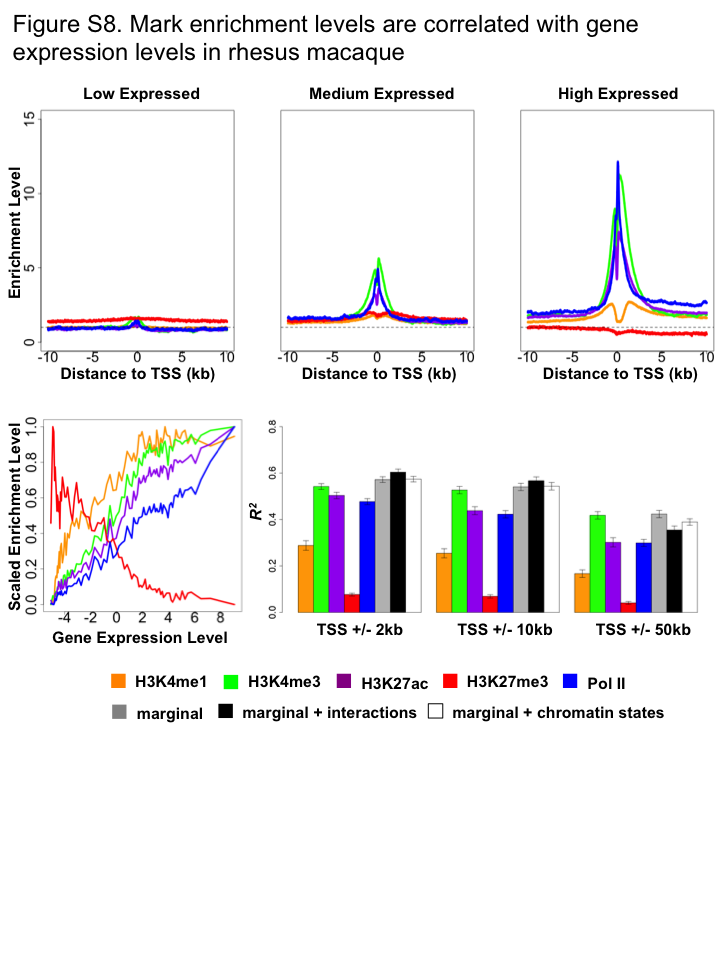

Supplement: Additional file 9: Figure S8. — Mark enrichment levels are correlated with gene expression levels in rhesus macaque. Legends are identical to those in Figure 2. [file 13059_2014_547_MOESM9_ESM.tiff]

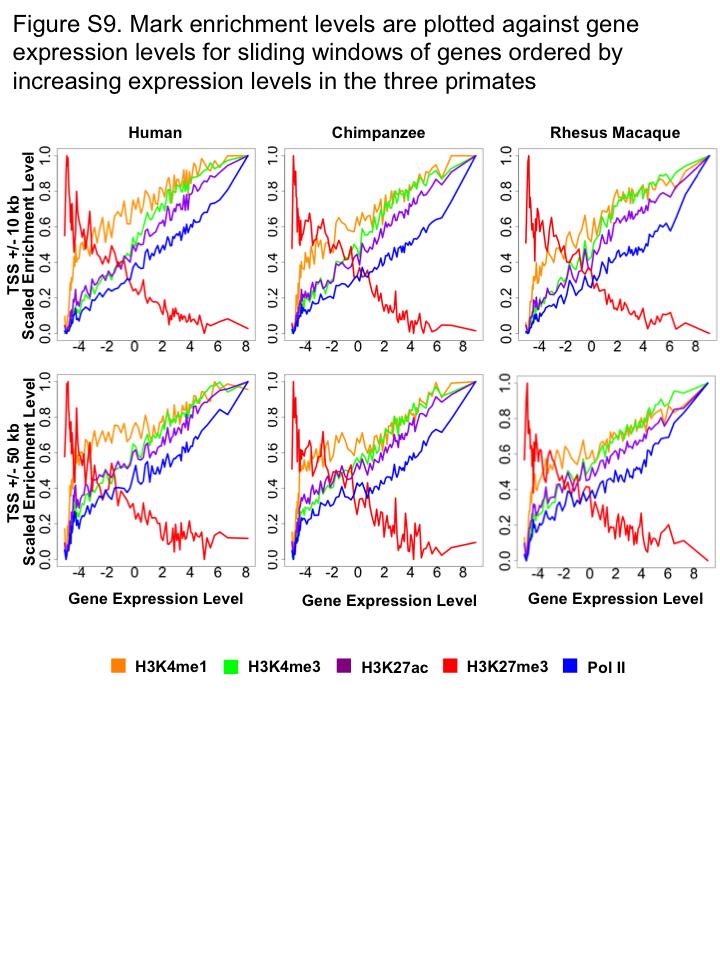

Supplement: Additional file 10: Figure S9. — Mark enrichment levels are plotted against gene expression levels for sliding windows of genes (n = 200) ordered by increasing expression levels in the three primates. Enrichment levels are obtained in either ±10 kb or ±50 kb regions near TSSs and scaled to be between 0 and 1. All values are averaged across individuals and across genes in the window. [file 13059_2014_547_MOESM10_ESM.tiff]

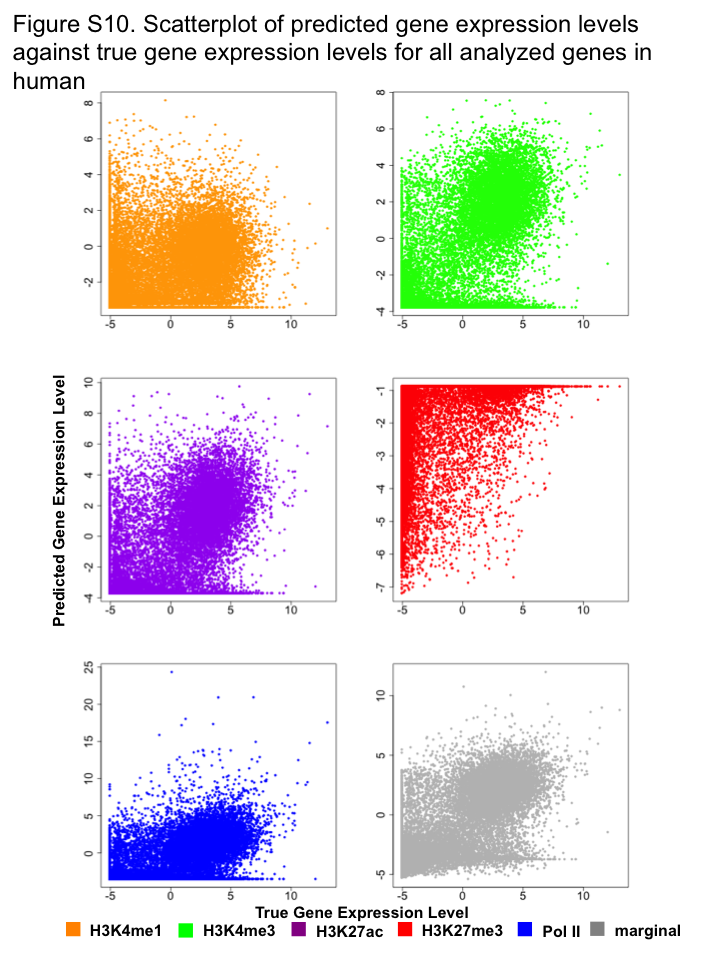

Supplement: Additional file 11: Figure S10. — Scatterplot of predicted gene expression levels against true gene expression levels for all analyzed genes in human. Predicted values are obtained based on linear models with either individual marginal effects (colored plots) or all marginal mark effects (grey plot) using mark enrichment levels in ±2 kb regions near TSSs. [file 13059_2014_547_MOESM11_ESM.tiff]

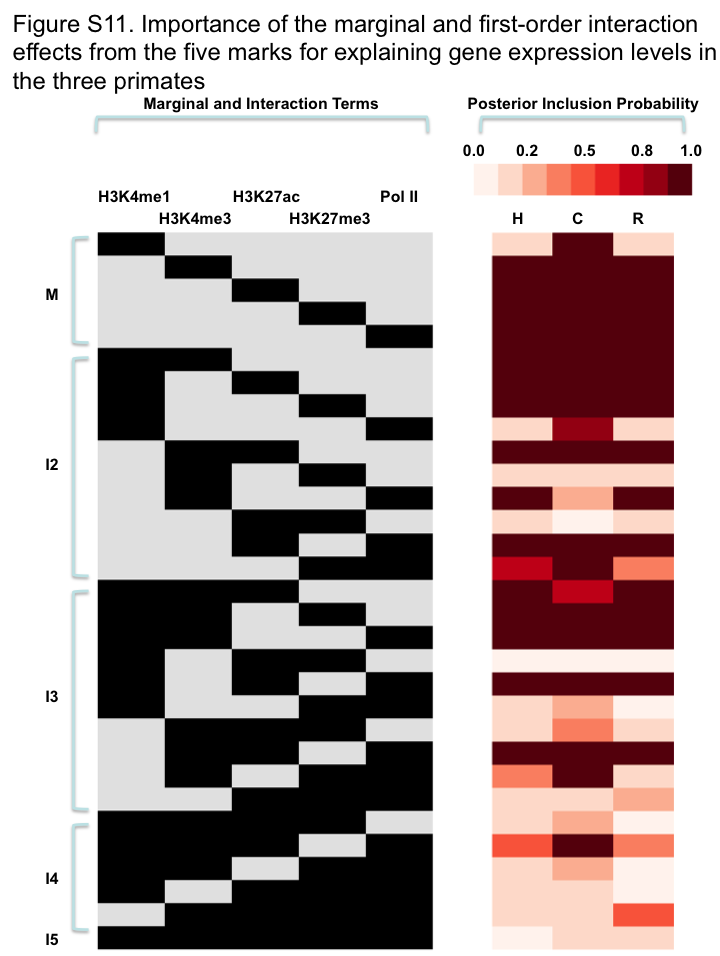

Supplement: Additional file 12: Figure S11. — Importance of the marginal and first-order interaction effects from the five marks for explaining gene expression levels in the three primates. The left panel lists all interaction terms among the five marks; each row represents an interaction term, and each column represents the presence (black) or absence (grey) of a particular mark effect for that interaction term. For example, the first row represents the marginal effect of Pol II, and the seventh row represents the interaction effect of H3K4me1, H3K4me3, and Pol II. The right panel lists the corresponding posterior inclusion probability of each term in the BVSR in the three species. The posterior inclusion probability measures the importance of each interaction term, with values ranging between 0 and 1; higher values indicate more importance. Mark enrichment levels ±2 kb regions near TSSs are used for fitting. C, chimpanzee; H, human; R, rhesus macaque. [file 13059_2014_547_MOESM12_ESM.tiff]

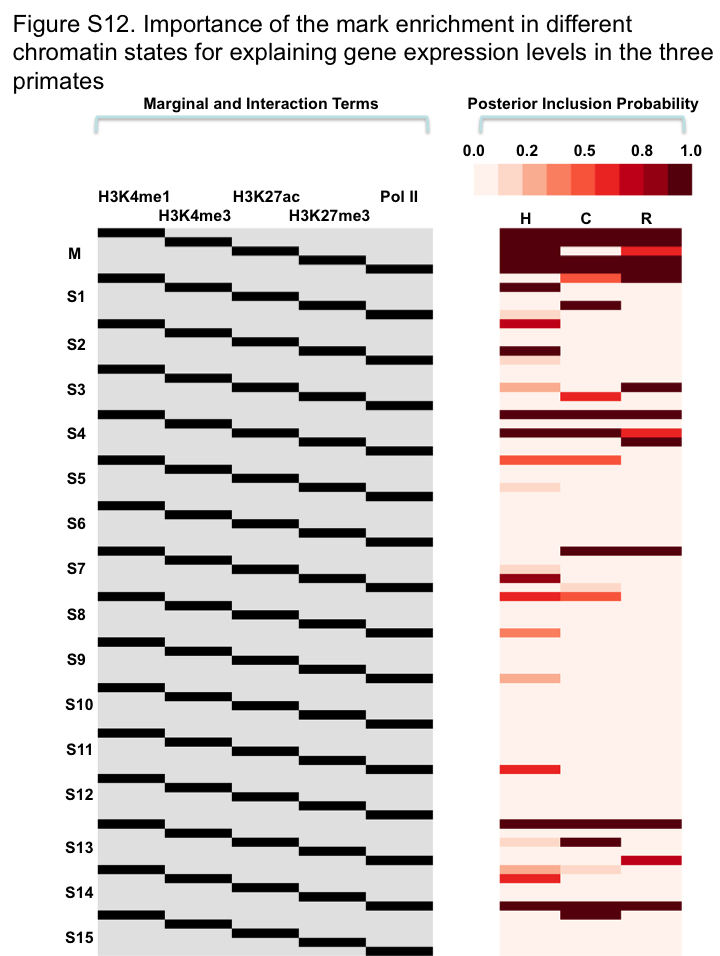

Supplement: Additional file 13: Figure S12. — Importance of the mark enrichment in different chromatin states for explaining gene expression levels in the three primates. The left panel lists marginal terms (M) or chromatin state-specific terms for 15 chromatin states (S1 to S15) near TSSs, where each column represents the presence (black) or absence (grey) of a particular mark effect for that term. For example, the first row represents the marginal effect of H3K4me1, and the sixth row represents the effect of H3K4me1 in chromatin state 1 (active promoter) near TSSs. The right panel lists the corresponding posterior inclusion probability of each term in the BVSR in the three species. The posterior inclusion probability measures the importance of each interaction term, with values ranging between 0 and 1; higher values indicate more importance. Mark enrichment levels ±2 kb regions near TSSs are used for fitting. C, chimpanzee; H, human; R, rhesus macaque. M, marginal effects; S1, active promoter; S2, weak promoter; S3, poised promoter; S4, strong enhancer; S5, strong enhancer; S6, weak enhancer; S7, weak enhancer; S8, insulator; S9, transcription transition; S10, transcription elongation; S11, weak transcription; S12, repressed; S13, heterochroma/lo; S14, repetitive/copy number variation; S15, repetitive/copy number variation. [file 13059_2014_547_MOESM13_ESM.tiff]

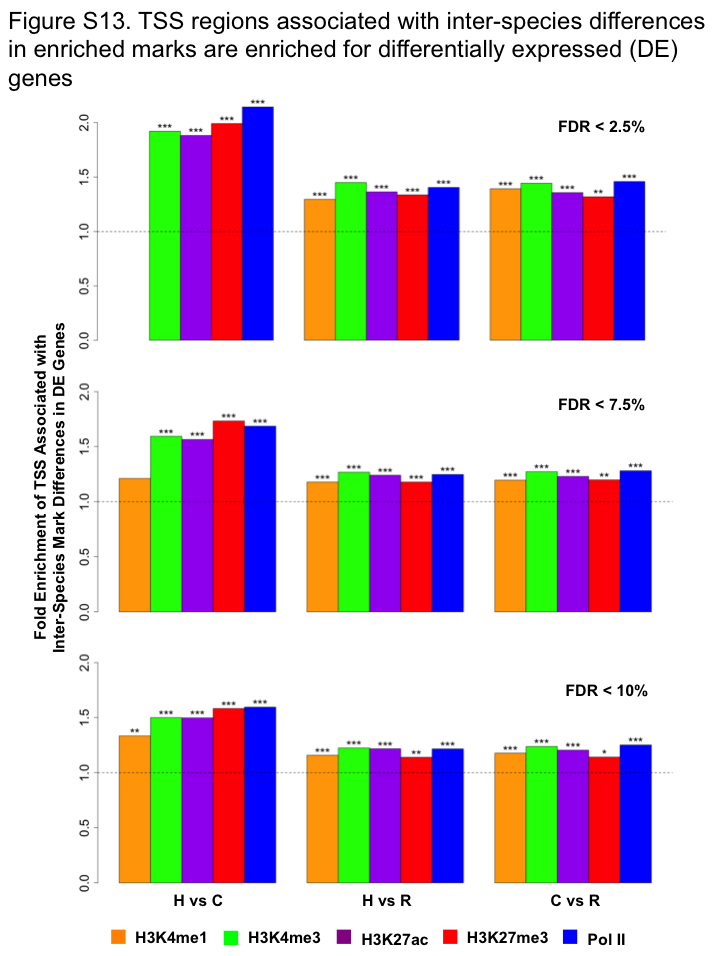

Supplement: Additional file 15: Figure S13. — TSS regions associated with inter-species differences in enriched marks are enriched for differentially expressed (DE) genes. TSS regions associated with inter-species differences in enriched marks and DE genes are determined by various FDR cutoffs (2.5%, 7.5%, and 10%). Legends are identical to those in Figure 3. C, chimpanzee; H, human; R, rhesus macaque. [file 13059_2014_547_MOESM15_ESM.tiff]

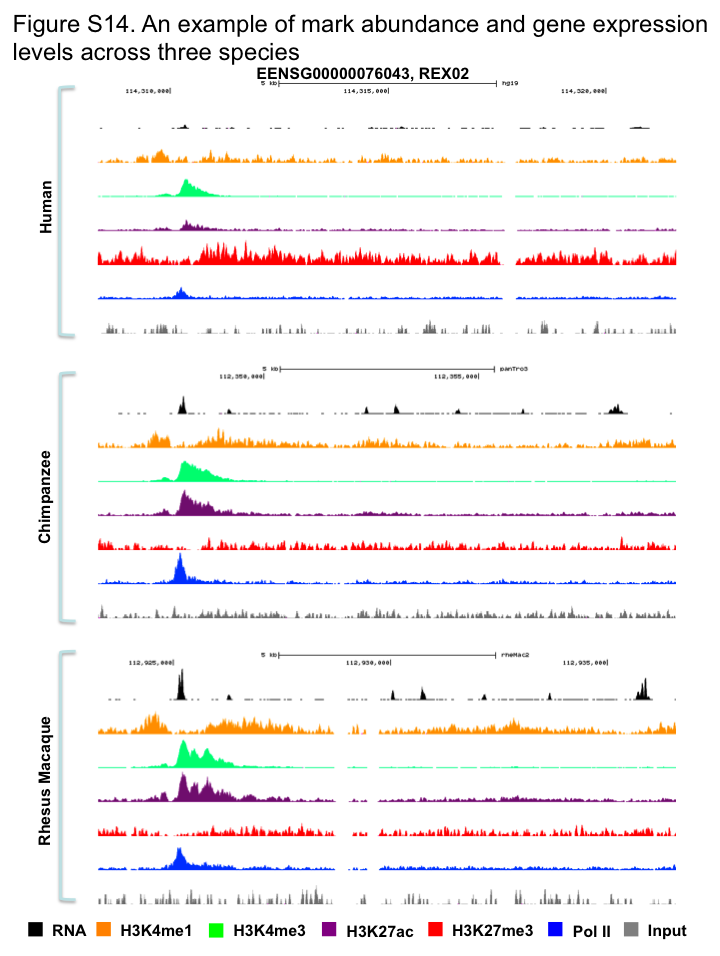

Supplement: Additional file 16: Figure S14. — An example of mark abundance and gene expression levels across three species. The x-axis is the distance along a genomic region containing the gene REX02. The y-axes show RNAseq reads (black), as well as ChIPseq reads for the five marks (color) and input controls (grey), all scaled with respect to the total mapped read counts. [file 13059_2014_547_MOESM16_ESM.tiff]

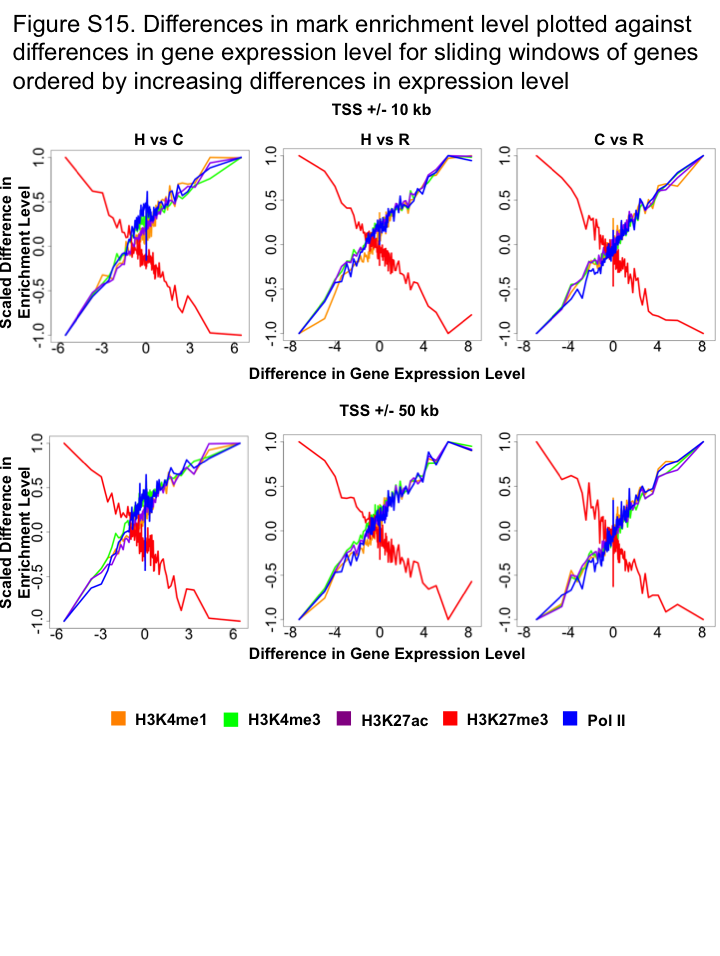

Supplement: Additional file 17: Figure S15. — Differences in mark enrichment level plotted against differences in gene expression level for sliding windows (n = 200) of genes ordered by increasing differences in expression level. Differences in enrichment level are obtained in either ±10 kb or ±50 kb regions near TSSs and scaled to be between -1 and 1. All values are averaged across individuals and across genes in the window. C, chimpanzee; H, human; R, rhesus macaque. [file 13059_2014_547_MOESM17_ESM.tiff]

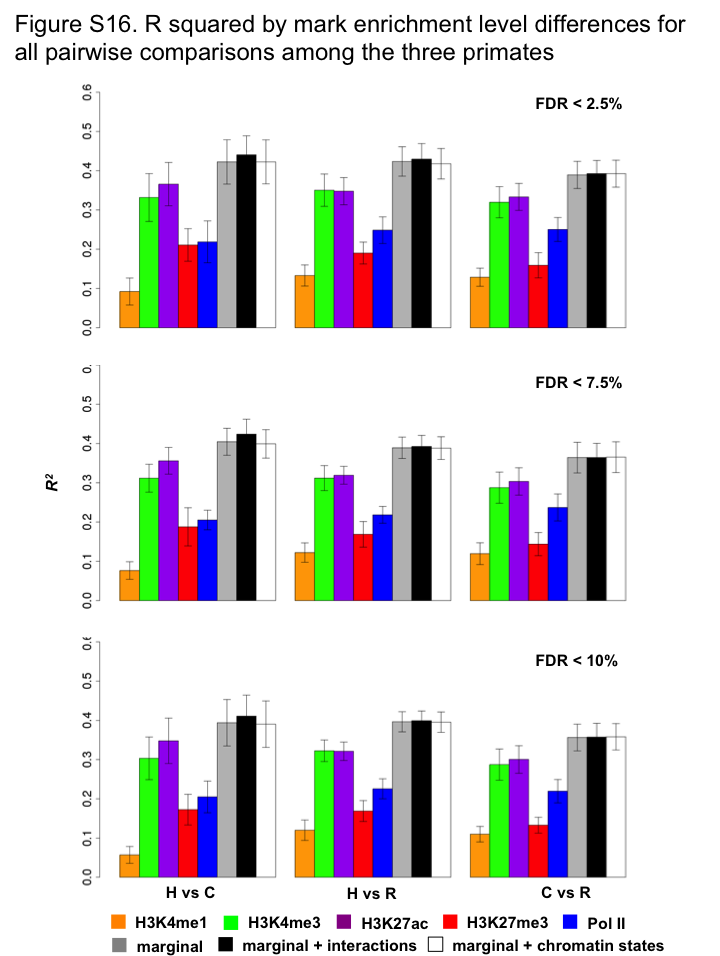

Supplement: Additional file 18: Figure S16. — Proportion of variance in gene expression level differences explained (R squared) by mark enrichment level differences, for all pairwise comparisons among the three primates. Different linear models are fitted to account for individual effects (five colored bars), combined marginal effects (grey bars) and all first-order interaction effects in addition to marginal effects (black bars), and all chromatin state-specific effects in addition to marginal effects (white bars) of the five marks. DE genes are determined based on an FDR cutoff of 5%. Enrichment level differences are obtained in ±2 kb regions. Error bars indicate standard deviation calculated across 20 split replicates. C, chimpanzee; H, human; R, rhesus macaque. [file 13059_2014_547_MOESM18_ESM.tiff]

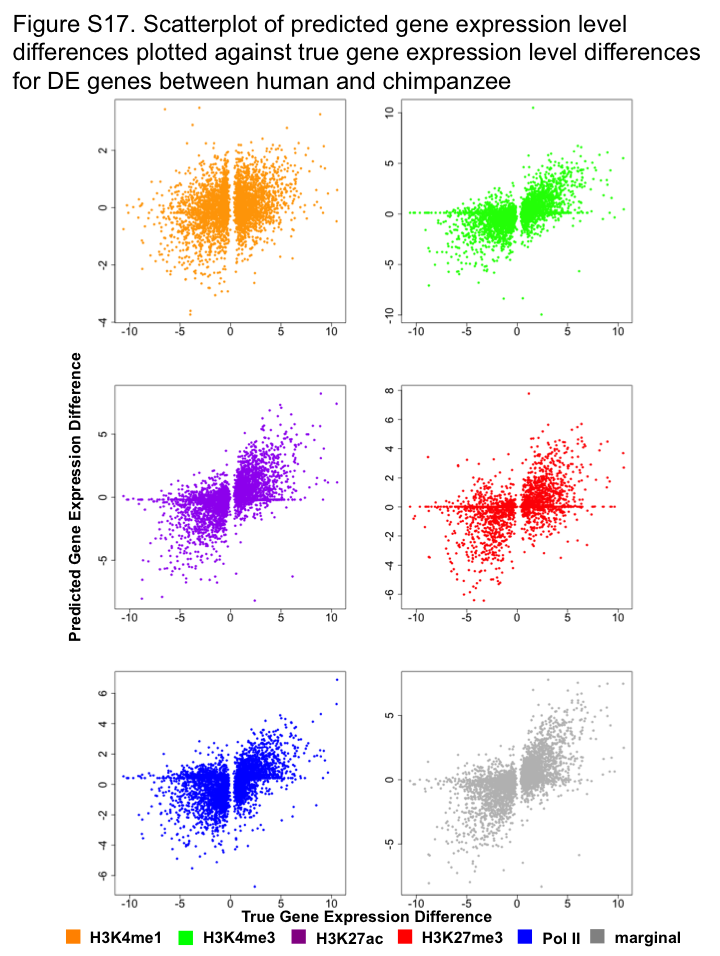

Supplement: Additional file 19: Figure S17. — Scatterplot of predicted gene expression level differences plotted against true gene expression level differences for DE genes between human and chimpanzee. Predicted values are obtained based on linear models using either individual mark effects (colored plots) or all marginal mark effects (grey plot) with mark enrichment level differences in ±2 kb regions near TSSs. DE genes are determined based on an FDR cutoff of 5%. C, chimpanzee; H, human; R, rhesus macaque. [file 13059_2014_547_MOESM19_ESM.tiff]

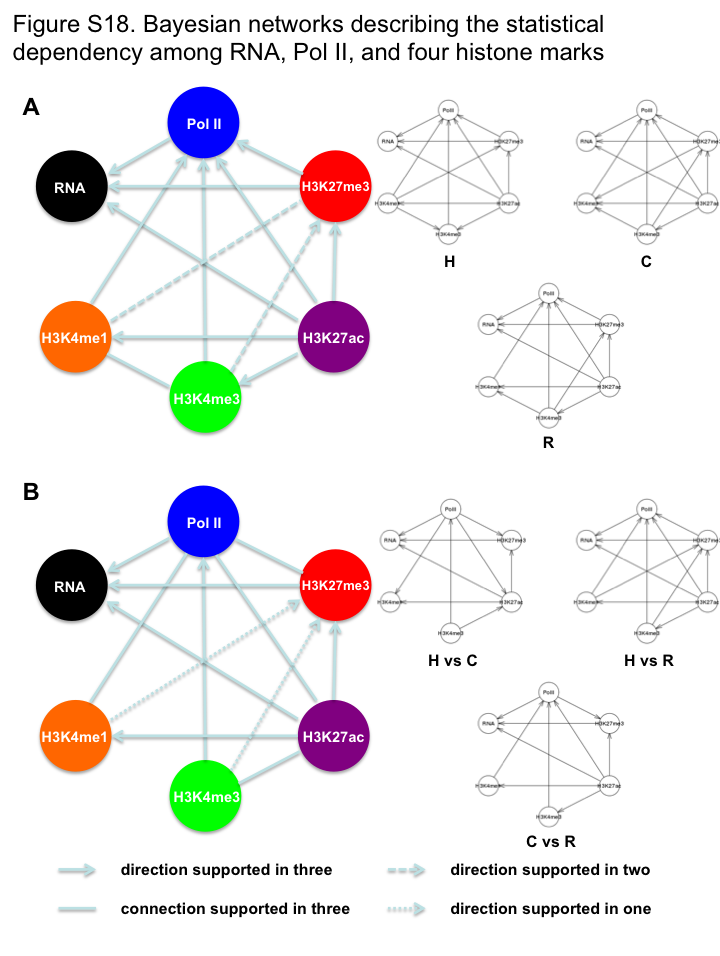

Supplement: Additional file 20: Figure S18. — Bayesian networks describing the statistical dependency among RNA, Pol II, and four histone marks. (A) A Bayesian network (left) describes the common statistical dependency among gene expression levels and mark enrichment levels, based on networks inferred from the three species separately (right). (B) A Bayesian network (left) describes the common statistical dependency among gene expression differences and enrichment level differences for the five marks, based on networks inferred from the three pair-wise comparisons (right). [file 13059_2014_547_MOESM20_ESM.tiff]

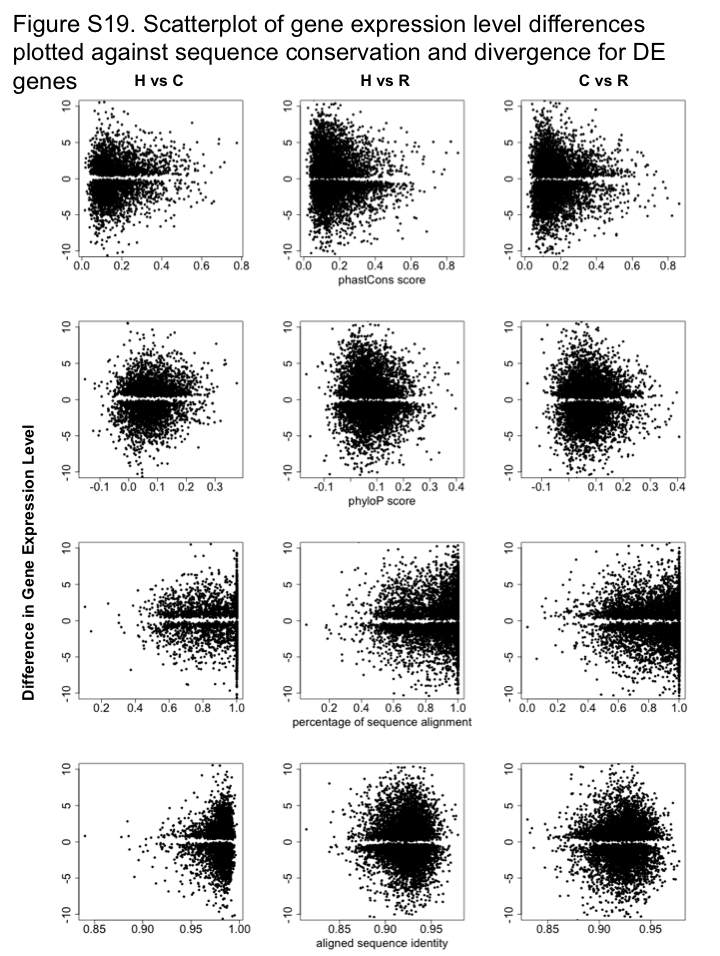

Supplement: Additional file 21: Figure S19. — Scatterplot of gene expression level differences plotted against sequence conservation and sequence divergence between pairs of species. Two sequence conservation measurements and two sequence divergence measurements are used. DE genes are determined based on an FDR cutoff of 5%. C, chimpanzee; H, human; R, rhesus macaque. [file 13059_2014_547_MOESM21_ESM.tiff]

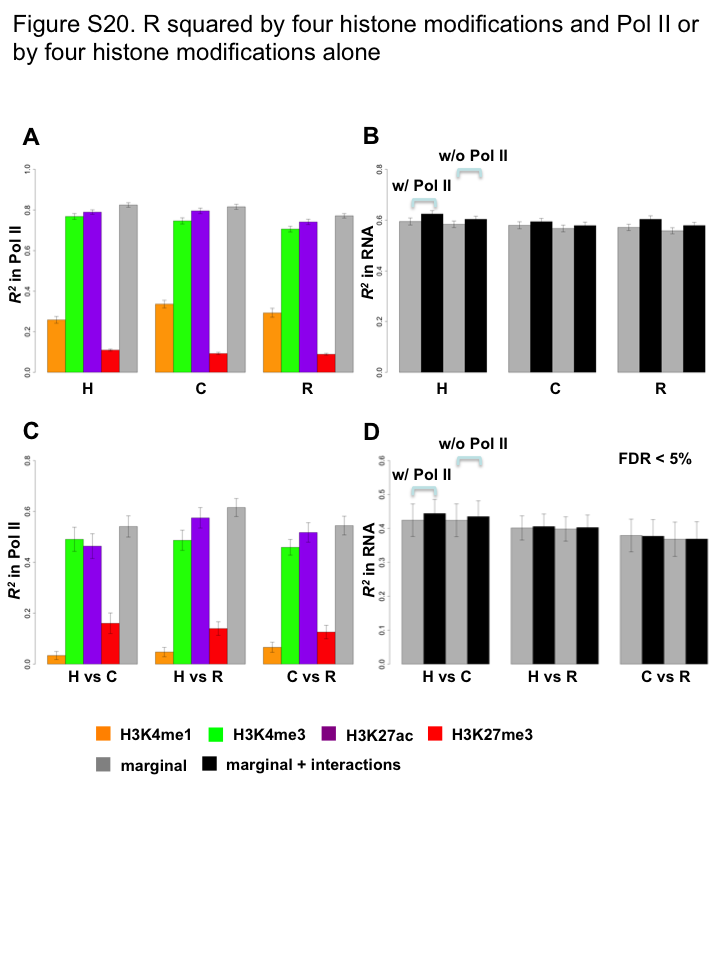

Supplement: Additional file 22: Figure S20. — R squared by four histone modifications and Pol II or by four histone modifications alone. (A) Proportion of variance in Pol II enrichment level explained by enrichment level of histone modifications. (B) Proportion of variance in gene expression level explained by mark enrichment level. (C) Proportion of variance in Pol II enrichment level differences explained by enrichment level differences of histone modifications in DE genes. (D) Proportion of variance in gene expression level differences explained by mark enrichment level differences in DE genes. Different linear models are fitted to account for combined marginal effects (grey bars) and all first-order interaction effects in addition to marginal effects (black bars). DE genes are determined based on an FDR cutoff of 5%. Enrichment level differences are obtained in ±2 kb regions. Error bars indicate standard deviation calculated across 20 split replicates. C, chimpanzee; H, human; R, rhesus macaque. [file 13059_2014_547_MOESM22_ESM.tiff]
